# The roles of highly conserved, non‐catalytic residues in class A β‐lactamases

**DOI:** 10.1002/pro.4328

**Published:** 2022-05-17

**Authors:** Aleksandra Chikunova, Marcellus Ubbink

**Affiliations:** ^1^ Leiden University Leiden The Netherlands

**Keywords:** BlaC, conserved residues, protein evolution, β‐Lactamase

## Abstract

Evolution minimizes the number of highly conserved amino acid residues in proteins to ensure evolutionary robustness and adaptability. The roles of all highly conserved, non‐catalytic residues, 11% of all residues, in class A β‐lactamase were analyzed by studying the effect of 146 mutations on in cell and *in vitro* activity, folding, structure, and stability. Residues around the catalytic residues (second shell) contribute to fine‐tuning of the active site structure. Mutations affect the structure over the entire active site and can result in stable but inactive protein. Conserved residues farther away (third shell) ensure a favorable balance of folding versus aggregation or stabilize the folded form over the unfolded state. Once folded, the mutant enzymes are stable and active and show only localized structural effects. These residues are found in clusters, stapling secondary structure elements. The results give an integral picture of the different roles of essential residues in enzymes.

## 1 INTRODUCTION

In the cause of evolution, organisms need to adapt to changing environmental conditions, so new enzyme functions must evolve, requiring changes in amino acid sequences. If such changes have a high chance of causing loss of function, the organism is less likely to adapt. Thus, evolution not only selects for functionality but also for evolvability. The potential to gain a new function is limited by the adaptability of the amino acid sequence. It is evolutionary disadvantageous to have many essential amino acid residues because mutations in these residues will often render a protein inactive. Therefore, evolution forces tend to minimize the number of essential residues.

Conservation of amino acid residues within or among protein families serves as a proxy for essentiality.^1^, ^2^, ^3^, ^4^ If an amino acid residue is highly conserved, it is assumed that evolution has selected against its substitution to prevent the loss of function of an enzyme.^4^, ^5^, ^6^, ^7^ Such a loss can have many causes, including impaired folding, enhanced misfolding, translocation problems, poor stability, enhanced breakdown, and poor function. Given all these possible causes of function loss, the number of highly conserved residues in enzymes is surprisingly low. Broadly, three groups of highly conserved residues can be distinguished, based on their location. The first‐shell residues are in the active site and involved in the catalytic function of the enzyme, so substrate binding and catalysis. Substitution of these residues will affect enzymatic function. Second‐shell residues surround the active site but are not directly involved in catalysis. It is assumed that these residues are important to ensure the exact positioning, to sub‐Ångström precision, of the first‐shell residues, which is required to enable catalysis of a chemical reaction. Third‐shell residues are farther from the active site and, thus, are less likely to affect activity, except in the case of allosteric interactions. Conservation of such residues is expected to be related to a role in folding, prevention of misfolding, and/or stability of the three‐dimensional structure.

**FIGURE 1:**
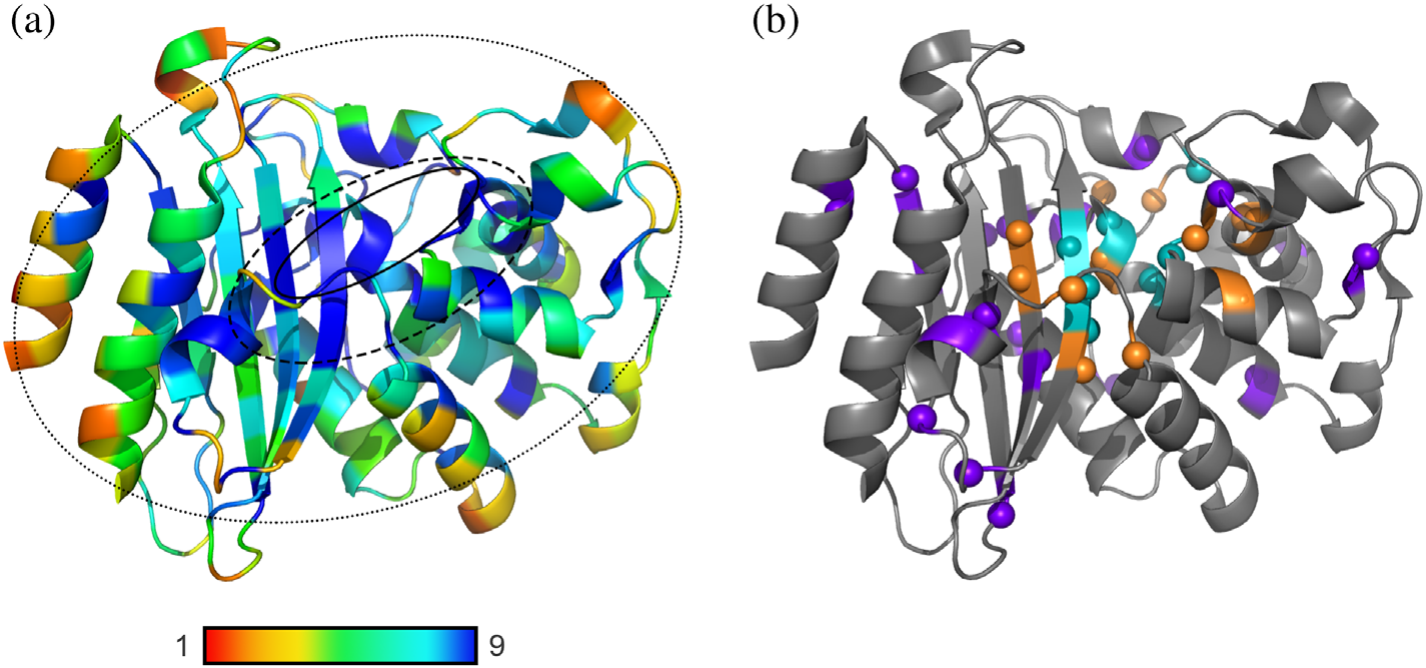
(a) Structure of β‐lactamase from Mtb (PDB entry 2GDN ^56^) colored by sequence conservation as determined by ConSurf^14^, ^15^, ^16^ with 1 being highly variable and 9 being highly conserved. Solid line, dashed line, and dotted line show the area of the first, second, and third shells, respectively; (b) Highly conserved residues (>92% with conservation score 9) in BlaC (PDB entry 2GDN). Residues of the first, second, and third shell are colored cyan, orange, or purple, respectively; backbone nitrogens are shown as spheres.

β‐Lactamases, enzymes responsible for the breakdown of β‐lactam antibiotics,^8^ exhibit high evolvability.^9^ Over the past decades, bacteria managed to respond to the high and constantly changing antibiotic selection pressures by evolving β‐lactamases able to degrade novel antibiotics. New β‐lactam resistant strains are frequently reported, with sometimes only one point mutation in the enzyme leading to a new catalytic function.^10^, ^11^, ^12^, ^13^ This room for evolution in β‐lactamases is reflected in the low amino acid conservation. A sequence alignment of the β‐lactamase from *Mycobacterium tuberculosis* (Mtb), BlaC, the object of this study, with 493 other class A β‐lactamases shows 49%–81% identity.^14^, ^15^, ^16^ Fifteen percent of the residues are extremely conserved, with the same residue type among more than 92% of the sequences. Figure 1 shows the degree of conservation mapped on the crystal structure of BlaC. Highly conserved residues are found not only in the active site but also widely spread over the three‐dimensional structure. While the roles of the conserved residues from the first shell have been studied extensively,^17^, ^18^, ^19^, ^20^, ^21^, ^22^, ^23^, ^24^ the residues from the other two shells received less attention.^25^, ^26^, ^27^, ^28^ In this study, our aim was to establish the roles of the highly conserved residues in the second and third shells of BlaC and to test the general idea that conserved second‐shell residues ensure the precise formation of the active site, whereas conserved third‐shell residues are important in folding and stability. Evolvability against new antibiotics and β‐lactamase inhibitors is related to the conservation of residues, so knowledge of which roles highly conserved residues play will aid in understanding evolutionary pathways toward new enzymatic functions.

We introduced single‐point mutations for all these conserved residues and characterized the activity, stability, and structure of the enzyme variants. The results of this study support the models formulated above and allow us to assign roles to the highly conserved residues. The structural analysis shows an interesting difference between second‐ and third‐shell residues in the extend of structural changes caused by the mutation. Surprisingly, for some variants folding, stability and activity are not affected, raising the question as to why these residues are conserved.

## 2 RESULTS

### 2.1 Several highly conserved residues can be mutated without activity loss

An overlay of the amino acid sequences of 494 class A β‐lactamases revealed 40 (15%) amino acid residues to be conserved in more than 92% of the sequences with the conservation score 9 (Figure 1, Table S1). Conserved residues in BlaC were divided into first, second, and third shells based on their proximity and association with the positioning of the active site (Figure 1). The first shell comprises eight conserved residues involved in catalysis and substrate binding. Residues that have at least one atom located within 4 Å of a first‐shell residue were considered the second shell and included eight residues. Residues that have at least one atom of their side chain within 4 Å of those second‐shell residues were also considered the second shell and counted four residues. The other 19 conserved residues that are located outside this sphere of direct influence on the positioning of the active site residues are defined as the third shell. The Cα of third‐shell conserved residues was found up to 25 Å away from the Cα of active site residue Ser70.

To establish the roles of the conserved residues in β‐lactamases, the second‐ and third‐shell residues were mutated separately to several amino acid types, and the variant enzymes were extensively characterized, both in cells, using an *Escherichia coli* expression system, and *in vitro*, using cell lysates with overexpressed BlaC. To make the number of mutants manageable, the variants were selected judiciously to vary the size, charge, and polarity of the side chain (Table S2), resulting in 146 variants for 31 highly conserved residues (11% of all residues) in the second and third shells. Two residues, at positions 132 and 164, were not included in the study, as BlaC is exceptional in that it carries non‐conserved amino acid residues at these positions.

**FIGURE 2:**
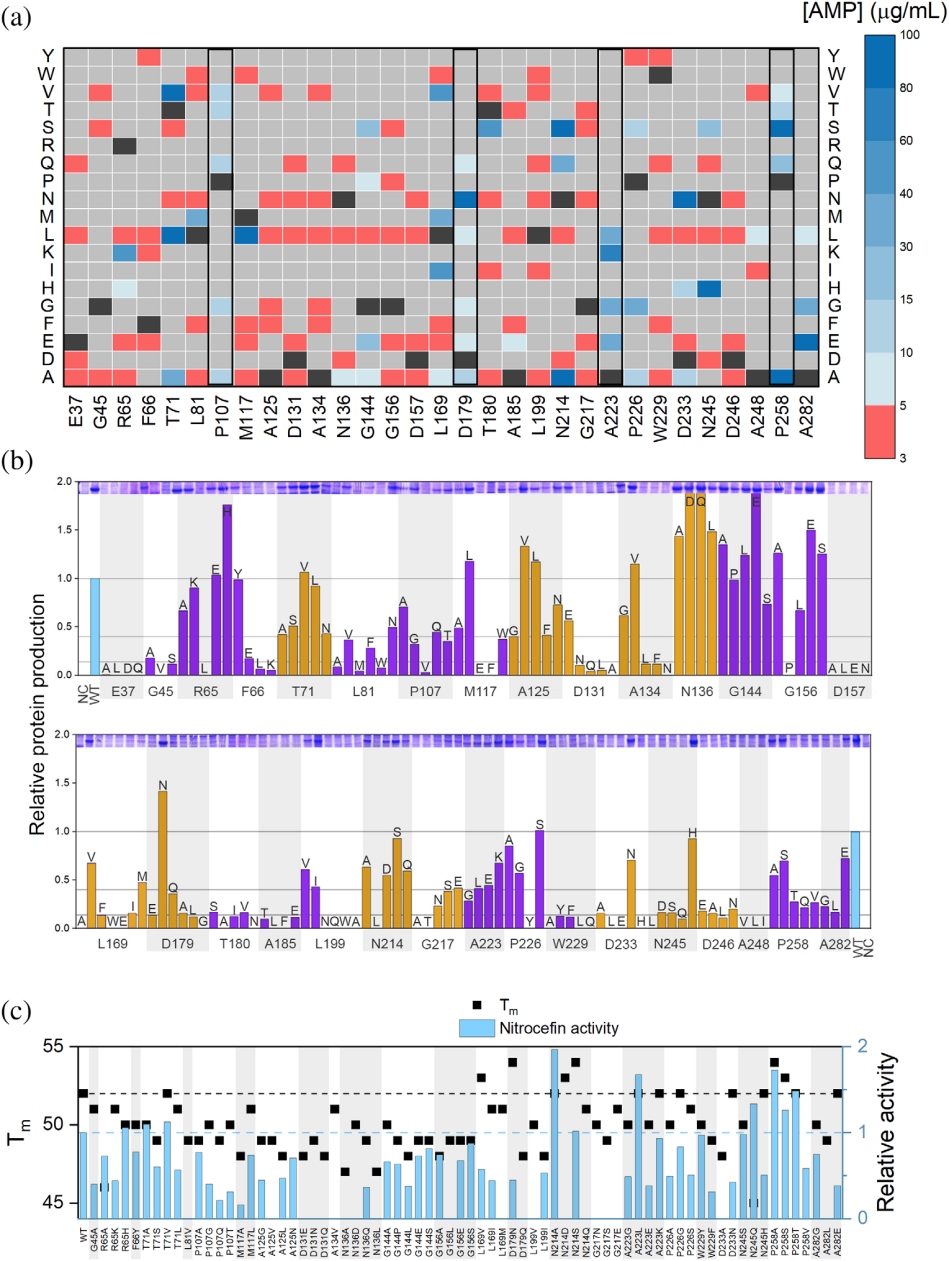
Activity, stability, and amount of soluble enzyme of BlaC variants. (a) MICs based on an ampicillin activity plate assay, with substitutions shown vertically. Wild‐type BlaC is indicated with a black box in each column and gray represents mutations that were not generated. Black rectangles show residues for which all generated mutations were tolerable; (b) Amount of BlaC found in soluble cell fraction of an *E. coli* cytoplasmic overexpression system relative to wild‐type BlaC, by comparison of the gel band intensity (shown on top of the histograms, full gels can be found in Figure S1). NC, negative control, the vector without the blaC gene. The third‐shell mutants are shown in purple; the second‐shell mutants are shown in orange. The horizontal bars indicate cutoffs for good/poor/no soluble protein production; (c) Melting temperatures and relative activity in nitrocefin conversion of soluble mutants in cell lysate. Horizontal dashed lines show the wild‐type BlaC values. The precision of the *T*_m_ is 0.5°. Mutants that did not yield folded enzyme sufficient for T_m_ and activity determination are not shown, mutants with a *T*_m_ but lacking an activity bar show no detectable activity for nitrocefin.

β‐Lactamase activity in cells was tested on LB‐agar plates with ampicillin and carbenicillin using a construct in which wild‐type BlaC is produced in low quantity and transported to the periplasm of *E*. *coli*, to mimic the location in Mtb.^29^ Compared to ampicillin, carbenicillin only carries an extra carboxyl group. Despite this subtle change in structure, minimal inhibitory concentrations (MIC) of ampicillin and carbenicillin differ by 10‐fold for wild‐type BlaC in *E. coli*, being 100 μg mL^−1^ and 1,000 μg mL^−1^ respectively. Most mutants exhibit decreased or no activity for both substrates (Figure 2a), but 12 mutants show activity comparable to that of wild‐type BlaC. Interestingly, these comprise mutations to another of the five amino acid groups (non‐conservative mutations), including T71V, T71L, N214A, N214S, and N245H, and some positions were able to tolerate all mutations that were introduced. Moreover, mutants D179N and N245H displayed MICs higher than wild‐type BlaC.

### 2.2 Many of the conserved residues are critical to obtain folded protein

The enzyme production level in a cytoplasmic *E. coli* overexpression system was monitored using gel electrophoresis. Production levels of the mutants were found to be close to the wild type; however, for more than half of the mutants, most protein was not soluble. These mutants also show no or very low ampicillin resistance in the low‐level expression, periplasmic system (Figure 2, panels a and b, Figure S1), indicating that both systems are at least to some extent comparable. It is noted that the periplasmic system is based on Tat‐translocation, in which the folded protein is translocated toward the periplasm. Thus, low solubility is likely to affect also the quantity of enzyme in the periplasm. This finding suggests that almost half of the conserved residues have a role in correct folding or avoiding misfolding.

All soluble mutants were further analyzed for their fold, stability, and activity. Because of the overproduction systems, these assays could be performed on cell lysates, not requiring the purification of each separate enzyme. Circular dichroism (CD) spectroscopy was used to determine the amount of folded enzyme relative to a wild‐type BlaC cell lysate, and this value was used in determining the activity of the mutant relative to that of wild‐type BlaC. Example data are shown in Figure S2. The chromogenic substrate nitrocefin was used to assay activity (Figure S3). For some mutants, little or no activity was detected due to the protein being unfolded, which was confirmed with both CD and NMR spectroscopies. Interestingly, most mutants for which soluble protein was obtained showed nitrocefin activity comparable to that of wild‐type BlaC (Figure 2c). This was observed also for the mutations that greatly affected the amount of folded protein. Correct folding was hindered by the mutation, but once folded, the enzyme behaves mostly as wild‐type enzyme. For others, such as N136, all variants gave protein yields comparable to or better than that for wild‐type BlaC, yet each had lost activity, as expected for second‐shell residues. A thermal shift assay was used to evaluate the thermal stability of mutant proteins. The majority of mutants show a slight decrease of melting temperature of 2–3°C (Figure 2c, Figure S4). BlaC N245Q showed the largest decrease, by 7°C. Some mutations increased the thermostability slightly, and for most of these variants activities were close to that of wild‐type BlaC. This observation indicates again that, once folded, the enzyme resembles wild‐type BlaC, although many residues that are important for folding also contribute to the thermal stability of the folded enzyme.

### 2.3 Second‐shell variants cause more wide‐spread structural changes than the third shell

**FIGURE 3:**
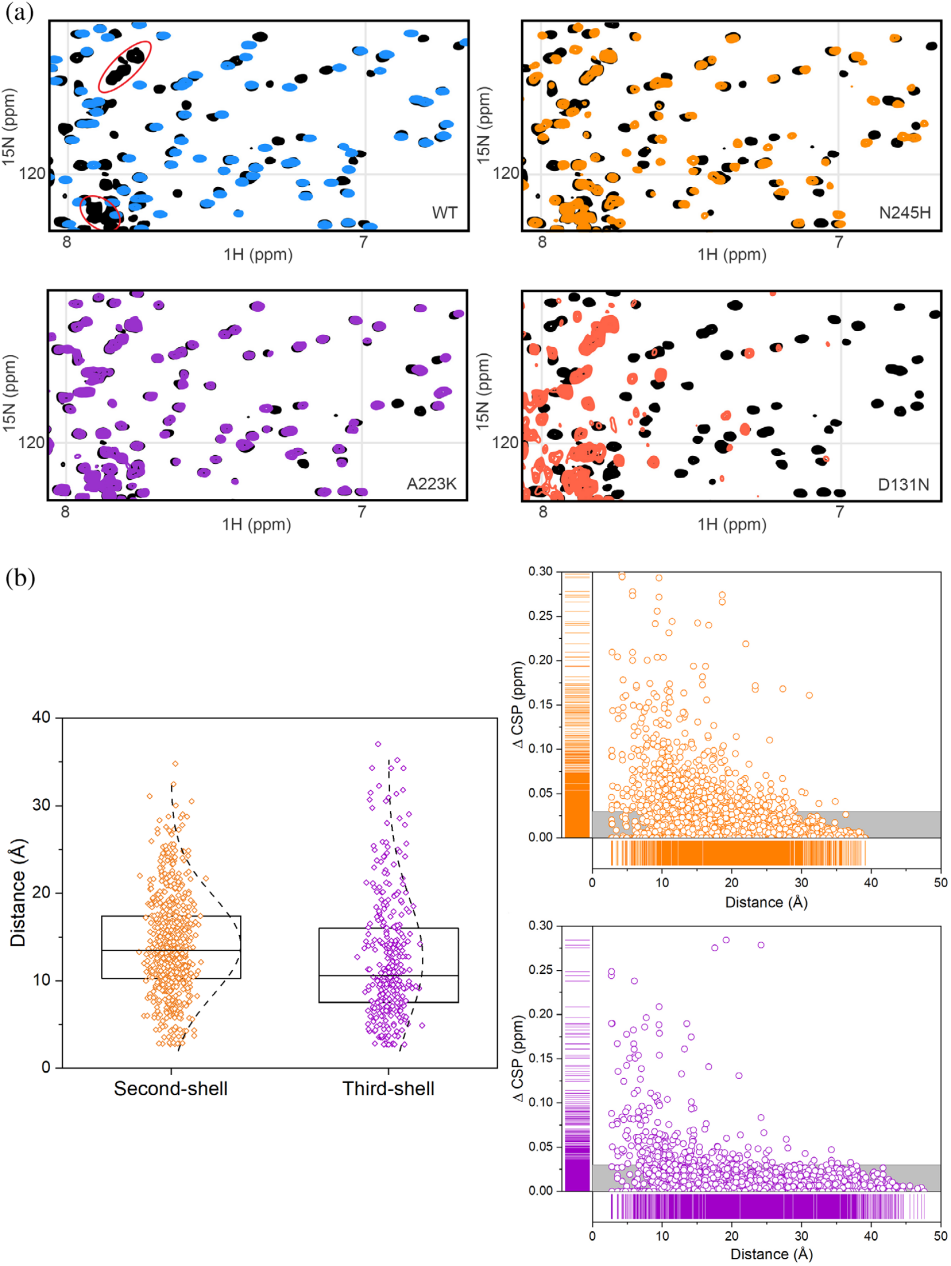
NMR TROSY spectra and CSPs. (a) Examples of details of NMR spectra (full spectra in Figures S5–S6). The wild‐type BlaC spectrum from the whole lysate is shown in black; purified wild‐type BlaC is shown in blue; spectra of well‐folded second‐shell (N245H) and third‐shell (A223K) mutants are shown in orange and purple, respectively; the spectrum of a poorly folded mutant (D131N) is shown in salmon. Red ovals indicate peaks from *E. coli* proteins. (b) All CSPs from all mutant spectra from the second (orange) and third (purple) shells are presented on the right against the distance from the mutation site (backbone amide to amide). Gray bars represent insignificant CSP (<0.03 ppm). On the left, CSPs above 0.03 ppm are shown for all assigned peaks of all mutants against the distance of the amide nitrogen relative to the Cα atom of the mutated residue. The boxes represent the 25th–75th percentile, lines inside the boxes represent the medians, and dashed lines represent distribution by count. The median of the second‐shell residues is significantly larger (*p* < 0.01) than that of the third‐shell residues.

**FIGURE 4:**
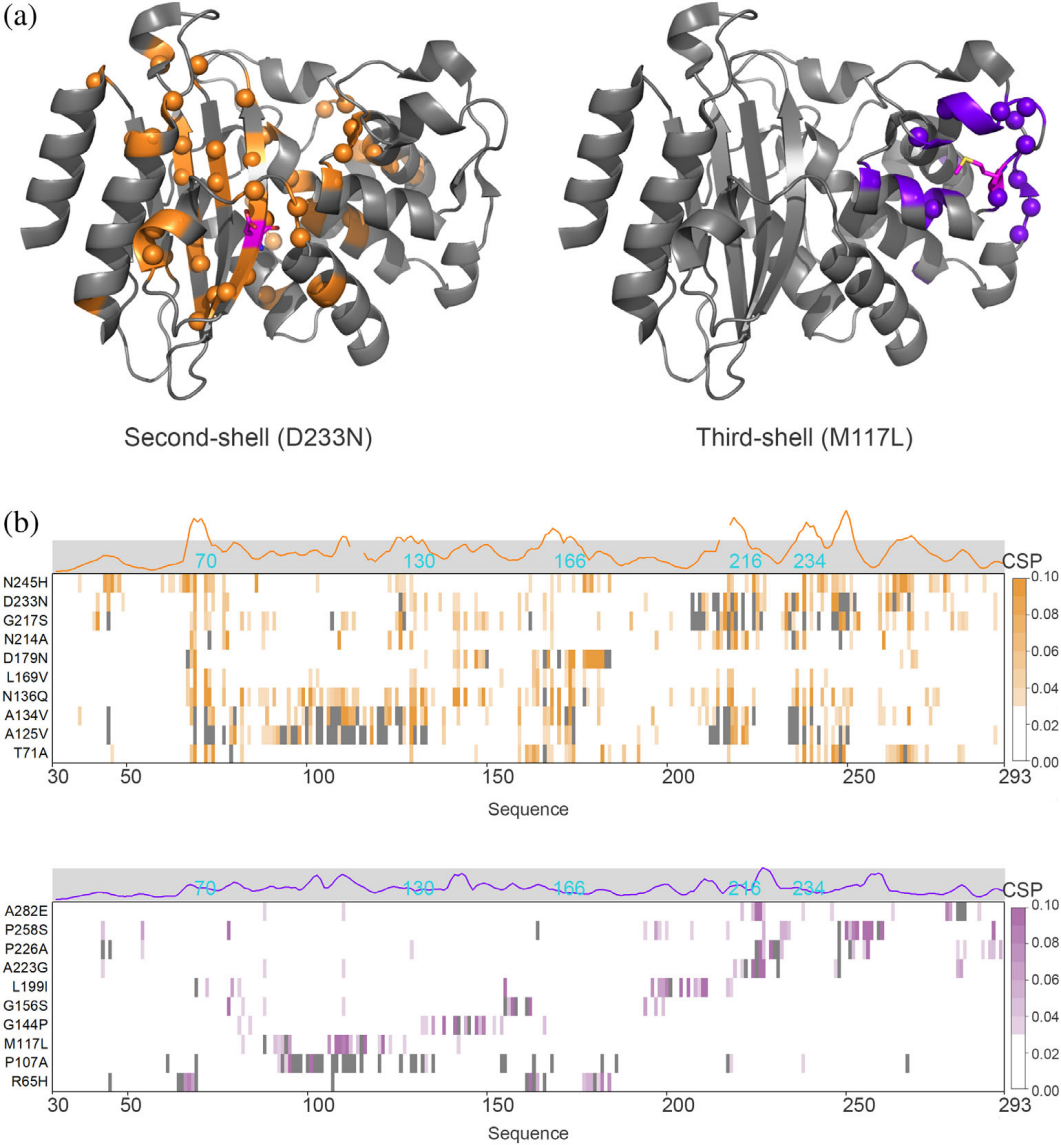
Changes in structure observed upon mutations. (a) Examples of the location of nuclei with significant CSP in second‐shell (left) and third‐shell mutants (right). CSPs are spread far from the mutation site in the second‐shell mutant D233N and are localized around the mutation site in the third‐shell mutant M117L. The mutated residue is shown in magenta sticks. PDB entry 2GDN.^56^ (b) CSPs of some mutants are plotted on the amino acid sequence. Residues for which no data are available are shown in gray. The positions of several first‐shell residues are indicated in cyan. For most second‐shell mutants, large CSPs are found for the same residues, in regions around residues 67–72, 110–112, 125–132, 162–169, 213–217, 232–238, 242–248, 261–266. Third‐shell mutants show CSP mostly close to the site of mutation. Traces above the graph represent averaged CSPs for all mutants, curves are smoothed using Savitzky–Golay filter to aid the visualization.

NMR TROSY spectra of the soluble fraction of the lysates were acquired to evaluate the fold of BlaC variants and the changes in structure that occur upon mutation (Examples in Figures S5–S6). For many soluble BlaC variants, the intensities of well‐dispersed peaks were low, pointing to a low amount of folded protein, and often intense signals in the region from 8.0 to 8.5 ppm indicated the presence of a fraction of unfolded protein (Figure 3a). For other mutants, the spectra were suitable for tentative peak assignment, which was done by comparison to a spectrum of wild‐type BlaC in lysate. Figure 3a shows an overlay of the spectra of His‐tagged enzyme in lysate and purified and His‐tag cleaved BlaC. Most resonances derived from BlaC nuclei can readily be identified.^30^ In general, NMR spectra of second‐shell mutants aligned worse with the spectrum of wild‐type BlaC than spectra of third‐shell mutants (Figure 3a). Significant chemical shift perturbations (CSPs), defined here as >0.03 ppm, were found for 17% and 6% of assigned peaks for second‐shell and third‐shell variants, respectively (Figure 3b). For second‐shell variants, CSPs were mostly found for the same set of residues, spread throughout and around the active site (Figure 4). The CSPs were also observed for mutants exhibiting only moderate change in activity or stability. For the third‐shell residues, CSPs were mostly localized around the mutation site. This interesting observation is general for all variants with good NMR spectra and suggests that the interactions that third‐shell residues make have low correlation with those further away in the protein (local structural effects only), whereas second‐shell mutations cause structural effects that are felt throughout the active site, showing correlation over a longer distance. The effects of the second‐shell mutations were similar for both core and surface localized residues (Figure S7). Only for 2 second‐shell residues in Ω‐loop, L169, and D179, CSPs were found to be spread somewhat less extensively than for the rest of the second‐shell residues; however, changes still involved the active site and regions around it (Figure 4b).

### 2.4 Conservative mutations can be more detrimental than non‐conservative ones

**FIGURE 5:**
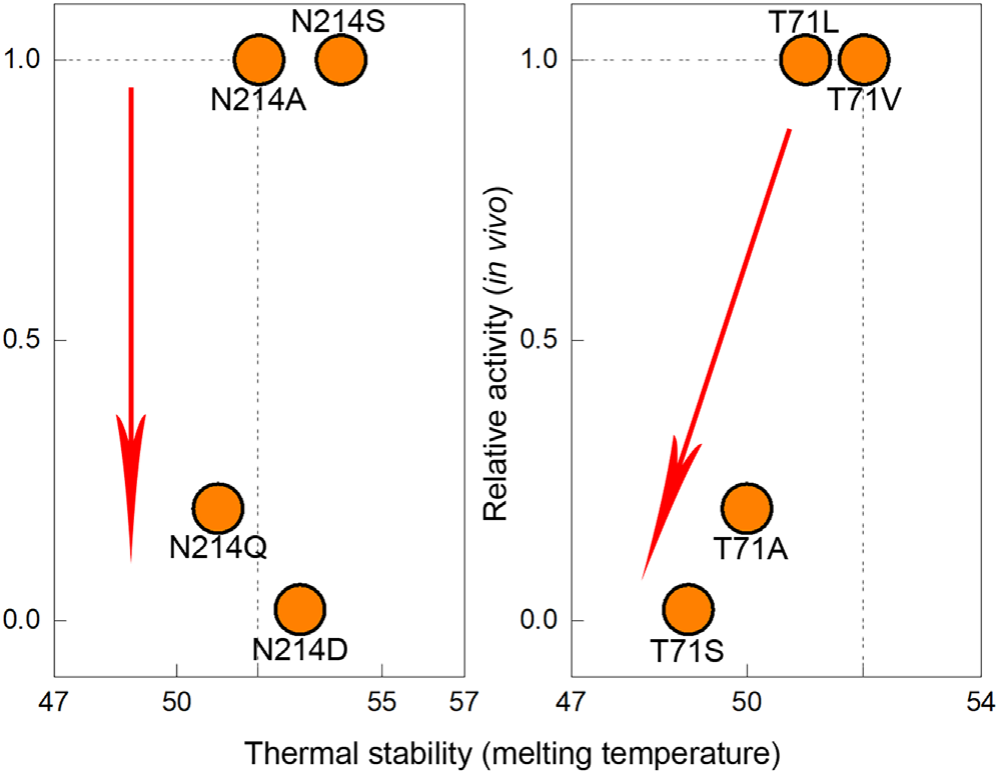
Schematic representation of the effect of mutations in residues Thr71 and Asn214 relative to WT. Dashed lines show the WT values. The red arrows show worsening of the mutation effect. For residue, Asn214 activity is affected more with conservative substitutions to Gln and Asp compared to non‐conservative Ala and Ser. For residue, Thr71 conservative substitution to Ser displays the worst activity and stability.

Conservative mutations of conserved third‐shell residues always had an equal or less detrimental effect on BlaC, as compared to non‐conservative mutations. For several variants of second‐shell residues, however, conservative mutations performed worse than non‐conservative ones, indicating that slight changes in the functional group have a more negative effect than replacement with a side chain that cannot maintain any interaction. Examples of such residues are Thr71 and Asn214. The conservative mutant T71S displayed in‐cell activity, thermal stability, as well as the quality of the NMR spectrum that was worse than those of T71 substituted by Leu, Val, or Ala. Mutants N214D and N214Q performed much worse in cells than mutants N214A or N214S (Figure 5, Table S2).

## 3 DISCUSSION

Many evolutionary studies have used β‐lactamases, especially TEM‐1, (e.g., Refs. [9, 31, 32, 33, 34]) because of their high adaptability and the ease to track changes in activity upon mutation. Directed evolution was carried out in numerous studies on various β‐lactamases of class A, including BlaC.^35^, ^36^, ^37^ However, directed evolution almost always yields substitutions in non‐conserved residues as they are the ones more likely to be mutated without negative consequences.^36^, ^37^ A few laboratory evolution studies on BlaC found mutations in the second‐shell residue 132, which is a conserved Asn in most class A β‐lactamases, but not in BlaC.^10^, ^17^ Deep mutational scanning was performed on TEM‐1, yielding the fitness effect of substitutions of every amino acid residue to all other 19 amino acids.^38^, ^39^ These extensive studies showed that robustness and evolvability of TEM‐1 are dependent on the strength of purifying selective pressure,^38^ and that mutational effects on protein thermodynamic stability shape the distribution of fitness effects of mutations.^39^ In these studies, the conserved positions are shown to be less tolerant to substitutions than the non‐conserved, which points to their essentiality. The work presented here aimed to further address this essentiality by assigning a possible role to the conserved residues.

**FIGURE 6:**
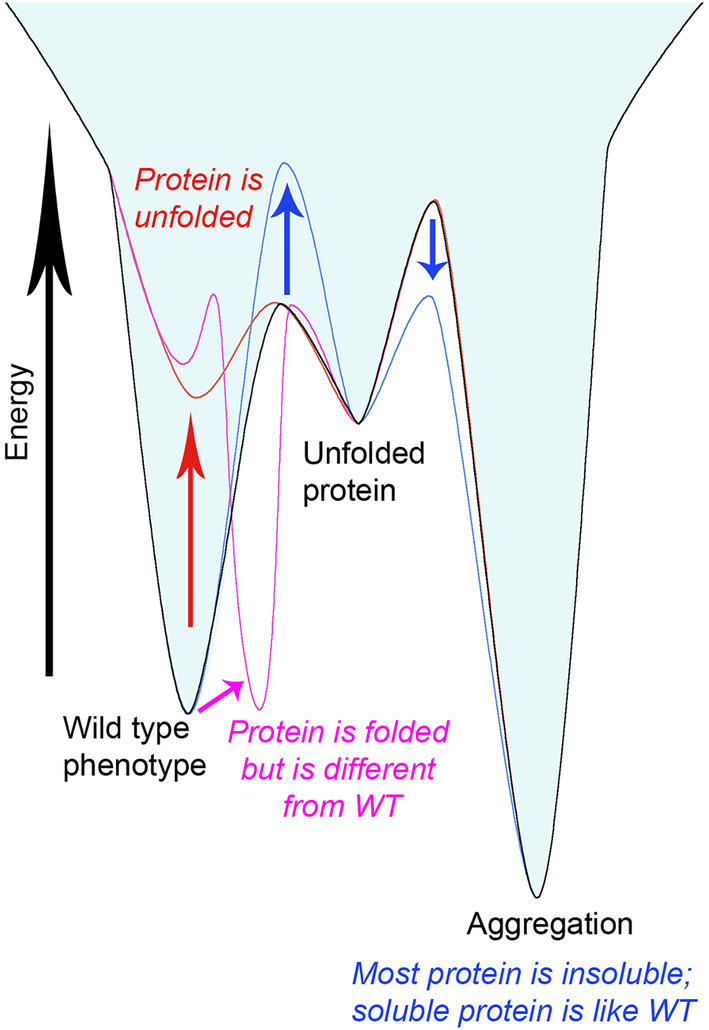
The free energy landscape of protein forms. The free energy landscape for wild‐type BlaC is shown with the black line. The effects of mutations described in the text for Groups A, B, and C are shown in blue, red, and magenta lines, respectively.

In general, we observed three types of mutation effects. Mutations in Group A result in strongly reduced amounts of soluble protein and proportionally more in the insoluble fraction. The proteins in the soluble fraction of these BlaC variants exhibit near normal stability, structure, and activity. These observations suggest that for these proteins, the mutation has shifted the balance between folding and formation of aggregates to the latter. Considering that folding into the soluble three‐dimensional structure is kinetically driven, it implies that the mutation either slows the folding rate or accelerates the aggregation rate (Figure 6, blue effect). Thus, these residues were most likely conserved by the evolutionary process to select against misfolding.^40^ The second group (B) concerns variants that yield substantial amounts of soluble protein of which a fraction remains unfolded, as demonstrated by the NMR spectra. The mutants in this group often have a reduced melting temperature. We hypothesize that in this case, the thermodynamic balance between folded and unfolded protein is affected. This implies that the conserved residue contributes to the stability of the folded form, relative to the unfolded state (Figure 6, red effect). The third group (C) is mutations that do not affect the production of a folded and stable enzyme but yield a non‐native form of the enzyme with reduced activity (Figure 6, magenta effect). We conclude that in this case, the conserved residue is critical to obtain the active conformation, within the ensemble of conformations that the enzyme can visit within the folded form. The major effect of mutations on protein production, stability of the native form, or activity was also demonstrated for other proteins. The mutational studies on two PER‐ARNT‐SIM domain proteins showed that many of the conserved residues contributed to the production of soluble protein.^41^, ^42^ Glutathione‐S‐transferase mutations in conserved motifs resulted in decreased stability and refolding rate, as well as in altered activity.^43^ The destabilizing effect of mutations in conserved positions was also demonstrated for acyl‐coenzyme A binding protein.^44^

**FIGURE 7:**
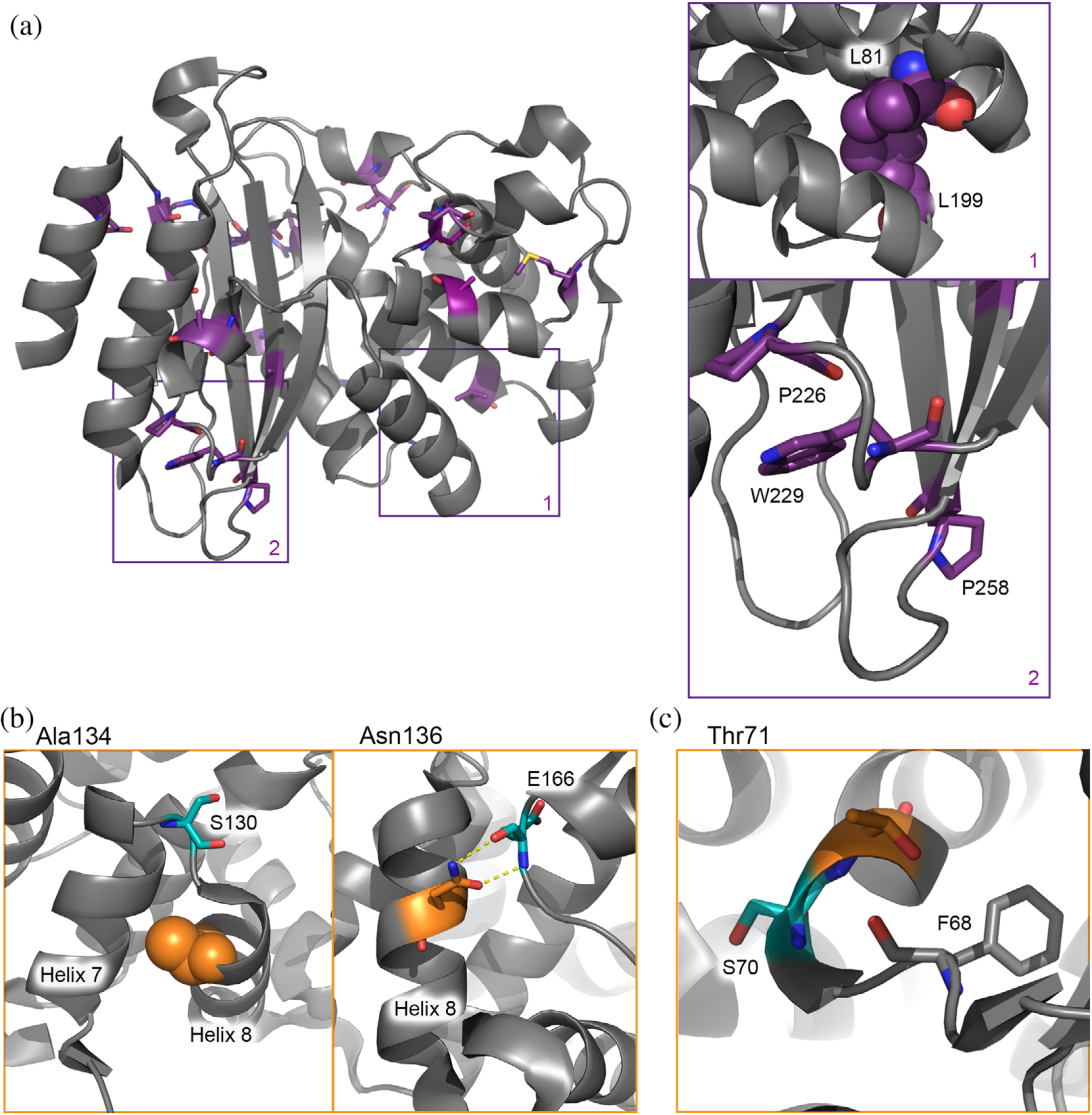
(a) Third‐shell residues localized around the edges of the secondary structure elements (shown in purple sticks or spheres) and zoom‐in of examples of interacting residues; (b) Examples of second‐shell residues (shown in orange) influencing the position of the first‐shell residues (shown in cyan) by ensuring the tight packing (Ala134) or making direct bonds to an active site residue (Asn136); (c) Environment of the second‐shell residue Thr71 (in orange).

The effect of Group A is mainly represented by conserved residues far from the active site (third‐shell residues). They are important for obtaining the correct three‐dimensional fold because, for many of them, mutation leads to a strong reduction in the amount of soluble protein (Figure 2, Table S2). Many are localized near the edges of the secondary structure elements (Figure 7a) and have interactions with nearby conserved residues, creating clusters. Such clusters are critical for the three‐dimensional structure by “stapling” the secondary structure elements together. Mutations diminish the folding efficiency and protein stability, but the fraction that is folded resembles the wild‐type enzyme structurally and kinetically.^25^ Protein stability and ability to fold are well‐known selection forces.^45^, ^46^, ^47^ Mutations elsewhere outside from the active site area in most cases are not detrimental as long as the secondary structure elements are formed and stapled by these essential residues. This principle results in a low number of essential residues outside the active site and thus a high evolutionary robustness of the protein. The effect of conserved residues clustering was shown for different folds,^48^ where such clusters play a role in ensuring the native form integrity of enzymes.

The Group B effect was shown for some second‐shell residue mutants. The soluble protein coexists with the unfolded form, which could be detected with NMR (Figure 3a, Figure S6). Highly rigid proteins tend to unfold slowly, and active sites have a destabilizing effect on proteins because they require a degree of dynamics or expose hydrophobic interaction sites.^49^, ^50^ Mutation of these Group B residues thus could increase the unfolding rate and shift the balance of folding and unfolding toward the latter by destabilizing the folded form.

The activity change observed for Group C mutants was evident to different degrees. Subtle effects (Group C1) were observed for a group of third‐shell residues containing glycines, prolines, and alanines, found mostly in solvent‐exposed loops (Table 1). These residues might be important for protein dynamics.^51^, ^52^ Very low activity (Group C2) was displayed by 12 well‐folded and stable mutants of second‐shell residues. These residues are located in places that ensure the exact positioning of the residues involved in catalysis. Examples of such residues are shown in Figure 7b. Residue Ala134 is located in α‐helix 8 and its side chain points toward α‐helix 7. Introducing a larger side chain is likely to interfere with the relative positions of these helices, dislocating active site residue Ser130. Residue Asn136 is also found in α‐helix 8, making two hydrogen bonds to backbone atoms of catalytic residue Glu166 and ensuring the cis conformation of the peptide bond between Glu166 and Pro167.^28^

**TABLE 1:** Effect of mutations and possible functions of conserved residues of the second and third shells.

Residue	Conservation among 494 sequences (%)	Other natural variants (%)	Effect of mutations (group)	Hypothetical role	Shell
E37	99.2	A, D, Q, S (0.2)	Mutations cause loss of in‐cell activity due to production of either insoluble or soluble unfolded protein; for some residues conservative mutations lead to production of soluble protein in low quantities, with a phenotype comparable to wild type. (A/B)	Ensures correct folding by priming the position of the first β‐strand^a^	III
G45	100			Allows the side chain of conserved residue F66 to occupy its position	III
R65	96.2	A (1.6), T, P (0.6), L (0.4), H, K, C (0.2)		Stapling function, stabilizes the position of Ω‐loop via interaction with conserved residue T180	III
F66	99.8	Y (0.2)		Locks β6 on β1 via interaction with residue 43	III
L81	99.2	M, V (0.4)		Is involved in hydrophobic interactions between α3, α7, and α13	III
M117	92.5	L (6.8), P (0.6), I (0.4)		Fills hydrophobic cavity between α6, α7, and α8	III
D157	99.2	N (0.8)		Is involved in stabilization of Ω‐loop	III
T180	99.6	S, I (0.2)		Stabilizes the Ω‐loop via interaction with conserved residue R65	III
A185	94.3	S (2.2), T (1.2), V, E (0.6), Q (0.4), R, N, G (0.2)		Fills small space between α12 and a loop, allowing for correct positioning of side chain of conserved residue D157	III
L199	100			Keeps bend between α12 and α13 inside the hydrophobic cavity	III
W229	98	S (0.8), A (0.6), Y, C, F (0.2)		Contributes many hydrophobic and stacking interactions	III
A248	74.5	G (25.5)		Fills small space inside the core of the protein	III
P107	98.6	A (0.8), V, Q, T (0.2)	Slight effect on stability and/or activity.(C1)	Is involved in a formation of a loop. Can be important for protein dynamics.	III
G144	99	D (0.6), S, N (0.2)		Is involved in a formation of a loop. Can be important for protein dynamics.	III
G156	97.8	N, D (0.6), E, S (0.4), R (0.2)		Makes a turn allowing for correct position of conserved residue D157	III
A223	97	K (1.2), V, S (0.4), E, R, M, T, G (0.2)		Points in the solvent together with conserved residue A282 might be involved in protein‐membrane interaction	III
P226	98.4	Q, A (0.6), L, D (0.2)		Is involved in stapling the turn between α13 and β7 via interaction with conserved residue W229	III
P258	98	A, S, T (0.6), Q (0.2)		Allows for a bend between β8 and β9	III
A282	94.2	E (1.3), S, T (1.1), K (0.9), G (0.7), V (0.4), R (0.2)		Points in the solvent together with conserved residue A223 might be involved in protein‐membrane interaction	III
A134	99.4	S (0.4), G (0.2)	*In vitro* activity loss with or without effect on folding.(C2)	Fills small space between α6, α7, and α8 together with conserved residue A125, allowing for correct positioning of active site Ser130	II
N136	100			Reduces mobility of the first‐shell residue E166 via two H‐bonds; stabilizes *cis* bond between E166 and P167^b^	II
G217	94.3	S (5), N (0.4), T (0.2)		Makes a turn aiding the correct position of the first‐shell residue T216	II
D246	96.4	N (1.4), T (1), A (0.6), I (0.4), E (0.2)		Pulls α14 to β8; might orient conserved residue D233 for its bond with conserved residue N214	II
T71	98.2	S (0.6), A, V, L (0.4)	Various effects on protein stability and/or activity; effect is dependent on type of substitution.(B/C)	Reduces mobility of the active site residue S70 via H‐bond to residue 68	II
A125	99.8	G (0.2)		Fills small space between α6, α7, and α8 together with conserved residue 134, allowing for correct positioning of active site Ser130	II
L169	95	I (2.6), M (1.6), V (0.8)			II
N214	92.9	S (3.6), T (1.4), C (1), A, G (0.4), V (0.2)		Stabilizes position of the first‐shell residue T216 via bond to a conserved residue D233; affects active site residue K234 via water	II
D233	96.8	E (2), G, H (0.4), S (0.2)		Interacts with conserved residues D246 and N214, influencing the first‐shell residues T216 and K234	II
N245	92.5	A (1.8), H, L, G (1.6), S (0.6), I (0.2)		Makes an interaction with residue 68, allowing it to position precisely for an interaction with conserved residue T71	II
D131	100		All mutations caused production of unfolded or partly folded protein.(B)	Plays role in protein stability, holds together α6, α7, and α8, positioning active site S130, via bonds to residues V108, A109, A125, T133, A134, A135^c^	II
D179	99.8	G (0.2)	Mutation D179N had a positive effect on stability, the other mutations affected protein folding and activity.(B/C)	Stabilizes the Ω‐loop via bonds with residues D172, D163, A164; might be involved in stabilization of active site S70 via bond with residue 68	II

^a^ Chikunova, A. et al. Conserved residues Glu37 and Trp229 play an essential role in protein folding of β‐lactamase. *FEBS J*. **288**, 5,708–5,722 (2021).

^b^ Banerjee, S. et al. Probing the non‐proline cis peptide bond in β‐lactamase from Staphylococcus aureus PC1 by the replacement Asn136 → Ala. Biochemistry **36**, 10,857–10,866 (1997).

^c^ Swarén, P. et al. Electrostatic analysis of TEM1 β‐lactamase: effect of substrate binding, steep potential gradients, and consequences of site‐directed mutations. *Structure* **3**, 603–13 (1995).

An interesting observation was made for a group of second‐shell residues for which the effect of the mutation is dependent on the nature of the new side chain. For these residues, variants with substitutions that remove the functional group are performing better than mutants with conservative substitutions. This observation emphasizes that the functional enzyme, especially around the active site, consists of a complex and extensive web of interactions. The presence of such a web is also supported by the NMR data, showing CSPs spreading far from the mutation site for the second‐shell residues. CSP can represent minute structural changes that are not easily picked up in crystal structures yet may have catalytic consequences if they cause small changes in the atom positions of catalytic residues or polarity changes in the active site. Mutations that influence the interactions of the side chain cause changes in the whole web of interacting residues and result in a dysfunctional protein. Substitutions that simply eliminate the interactions of the side chain apparently make it possible for other residues to compensate for the lost interaction. For example, residue Thr71 probably aids the precise positioning of catalytic residue Ser70, by providing a H‐bond between the Oγ1 and the carbonyl oxygen of Phe68 (Figure 7c). Substitution T71S, which is likely to modify the length of this H‐bond, yields less stable protein with somewhat decreased activity against nitrocefin *in vitro* and no activity against ampicillin and carbenicillin in cells. However, substitutions to Leu, Val, or Ala, which eliminate this H‐bond completely, result in protein that resembles wild‐type BlaC better.

All analyzed residues had conservation higher than 92%. Such high conservation of a residue suggests that the exact nature of the side chain is extremely important. Nevertheless, a few mutants demonstrated behavior close to that of wild‐type BlaC. Two mutants, BlaC N245H and D179N, exhibit in fact in‐cell activity higher than wild‐type BlaC, accompanied by a melting temperature that is the same or higher. One would expect to find these residues in natural variants of β‐lactamases as often as the conserved residues, yet the extreme conservation shows the preference toward the native residue. A few explanations can be given for this phenomenon. First, our experiments could only characterize a limited set of protein properties, activity against two specific antibiotics and nitrocefin, protein production, thermal stability, and structural changes. It is possible that these mutants have lost a trait not tested within this research. A second explanation is that not all amino acid substitutions can appear equally often. The transition/transversion bias can contribute to the lower chance of some mutations to occur in nature.^53^ One example of such substitution can be the mutant N245H, which requires a change from adenine to cytosine. Furthermore, some amino acid substitutions require two nucleotide changes. For example, substitutions of T71V or T71L that happen via consecutive point mutations can only occur via T71P, T71A, T71M, or T71I. We have no data about the effects of T71P, T71I, and T71M on the protein, but based on the position of Thr71, it is possible to assume that at least substitutions to Pro and Met will have a negative effect on the protein function. A third explanation is that a residue is essential in most β‐lactamases but not in BlaC. For example, the variant D179N outperforms wild‐type BlaC in terms of in‐cell activity, stability, and protein level (*in vitro* activity is somewhat reduced). However, in TEM‐1, this variant shows reduced fitness against ampicillin selection pressure.^38^, ^54^, ^55^ Thus, it can be that Asp179 is essential for many class A β‐lactamases but for BlaC, so a mutation to Asn would not be detrimental, perhaps even an improvement for the fitness.

The results of this study led us to formulate a hypothetical function for each conserved second‐ and third‐shell residues in BlaC (Table 1).

In conclusion, this work offers an explanation of general trends of conservation in serine β‐lactamases. Conserved residues of the first shell form a catalytic center of a protein, conserved residues of the second shell establish a functional core of the protein and the third‐shell residues ensure the overall fold. For the third‐shell residues, the interactions they make in a final form of a protein or during the folding process are extremely important. For that reason, many of them do not tolerate any mutations, and others can only undergo conservative substitutions. Residues of the third shell contribute to a high evolutionary robustness of the protein by limiting conservation to a few clusters that are essential to staple secondary structure elements correctly. The second‐shell residues form a web of interactions around the active site, fine‐tuning the structure of the active site. In that way, residues of the second shell contribute to a high evolvability of the protein. The conserved residues of the second shell ensure the overall integrity of the active site, while random mutations can accumulate around the active site, potentially leading to a new trait.

## 4 MATERIALS AND METHODS

### 4.1 Conservation analysis

Sequence conservation among β‐lactamases was determined using the ConSurf server.^15^ The BlaC sequence from Wang, Cassidy, and Sacchettini^56^ (PDB ID: 2GDN) was used to find homologs using a PSI‐BLAST algorithm with five iterations. The minimum sequence identity was set to 35%. Sequence redundancy was reduced by CD‐HIT,^57^ with a maximum sequence identity of 95%. An alignment of 494 sequences^58^ was generated using the MAFFT algorithm^59^ and the level of conservation of each amino acid residue position was calculated.

### 4.2 Mutagenesis

Site‐directed mutagenesis was performed using two vectors as template. For in‐cell studies, mutants were created in a pUK21 (pUC21 based plasmid with kanamycin resistance) plasmid carrying the *blaC* gene, encoding a Tat signal peptide for transmembrane transport.^29^, ^60^ The plasmid pET28a  carrying the *blaC* gene with the code for a cleavable N‐terminal His(6)‐tag^61^ was used as overproduction system and to determine the amount of soluble protein. Mutagenesis was performed using the QuikChange (Agilent Technologies, Santa Clara, CA) method. The presence of the mutations was confirmed by sequencing.

To establish the localization of the mature protein, BlaC WT with the signal peptide for Tat‐system was overexpressed in pET28a  vector confirming that the protein was in membrane, while most protein was found in the cytoplasm for wild‐type BlaC expressed without Tat‐system signal peptide (Figure S8).

### 4.3 In‐cell activity studies

The survival of the *E. coli* cells carrying pUK‐based plasmids with wild‐type or mutant *blaC* genes was tested on LB‐agar plates with antibiotics. All plates contained 50 μg mL^−1^ kanamycin and 1 mM isopropyl β‐D‐1‐thiogalactopyranoside (IPTG). Cells were plated as 10 μL drops with OD_600_ values of 0.3. In the first round of in‐cell studies, the plates contained ampicillin (3 μg mL^−1^ or 15 μg mL^−1^) or carbenicillin (20 μg mL^−1^ or 200 μg mL^−1^). Mutants that did not grow on the lowest concentrations of antibiotics were not used in the following test rounds; the growth of mutants surviving the lowest concentrations but not the highest was tested on intermediary concentrations of ampicillin (5 μg mL^−1^ and 10 μg mL^−1^) and carbenicillin (50 μg mL^−1^ and 100 μg mL^−1^). For mutants surviving the highest concentrations, growth was tested on plates with 30, 60, and 100 μg mL^−1^ ampicillin and 500, 1,000, and 2000 μg mL^−1^ carbenicillin. All tests were performed with two biological replicates. In case of disagreement between replicates, mutants were tested separately again. *E. coli* cells expressing the gene of BlaC S70A were used as a negative control because these cells produce intact but inactive BlaC.

### 4.4 BlaC presence in E. coli *cells*

For recombinant production of mutant proteins, *E. coli* strain BL21pLysS (DE3) was used in combination with the pET28a  based plasmids. Cells were cultured in LB medium at 37°C until the OD_600_ reached 0.6–1.0, at which point protein production was induced with 1 mM IPTG, followed by incubation of the cultures at 18°C for 16 h. Of overnight bacterial cultures, 100 μL was tested for nitrocefin activity and 500 μL was centrifugated and resuspended in 50 μL B‐PER (Thermo Scientific, Waltham, MA) for lysis. After 30 min incubation at room temperature, 20 μL was treated with SDS‐PAGE cracking buffer (20 mM Tris/HCl pH 6.8, 5 mM EDTA, 0.5% SDS, 0.1% β‐mercaptoethanol), and the rest was centrifugated for separation of soluble and insoluble fractions. The soluble fraction was tested for nitrocefin activity and treated with SDS‐PAGE cracking buffer. Whole lysate and soluble fraction samples were analyzed using SDS‐PAGE and gels were stained with Coomassie Brilliant Blue. The signal intensity of the band corresponding to BlaC for each mutant was compared to the signal intensity of the *E. coli* protein at 40 kDa using Image Lab software (Bio‐Rad, Hercules, CA). The experiment was done with four replicates, and the gels were compared to each other. Only one replicate was used to calculate the relative expression.

Mutants that showed a clear band on the gel for the soluble fraction were analyzed *in vitro*. Cultures of 10 mL were induced and incubated overnight at 18°C and lysed with B‐PER for 30 min at room temperature. After centrifugation, the supernatant containing the soluble protein fraction of the lysate was diluted 100‐fold.

### 4.5 Thermal stability

The thermal stability of the proteins was analyzed with a thermal shift assay with SYPRO Orange dye (Invitrogen, Waltham, MA). The measurements were performed in triplicate using the CFX 96 Touch Real‐Time PCR Detection System (Bio‐Rad, Hercules, CA) with the temperature range of 20–80°C. Melting temperatures were determined from the averaged signal of three measurements. The technical error is 0.5°C.

### 4.6 Circular dichroism

CD profiles were recorded at 25°C using a Jasco J‐815 spectropolarimeter with a Peltier temperature controller (Jasco, Tokyo, Japan). Spectra were acquired in a 1‐mm quartz cuvette at a scan rate of 50 nm/min. The negative control spectrum was used as a background spectrum of *E. coli* proteins and subtracted from all mutants and wild‐type spectrum (Figure S2). The CD signal at 222 nm was then used as a measure for the amount of folded BlaC for calculating the activity of mutants relative to wild‐type BlaC.

### 4.7 Kinetics

Determination of the Michaelis–Menten kinetic constants was done by measuring the absorption change at 486 nm for nitrocefin (Δε = 25.7 mM^−1^ cm^−1^ based on concentration calibration using quantitative NMR against 2.0 mM trimethylsilylpropanoic acid) with an Infinite® M1000PRO plate reader (TECAN, Mannedorf, Switzerland) thermostated at 25°C. All measurements were performed in triplicate. Initial rates of the hydrolysis were plotted against the concentration of substrate, and Michaelis–Menten curves were fitted to the Michaelis–Menten Equation 1 using OriginPro 9.1,

(1) $$\upsilon_{0}=\frac{V_{\max}{\left[S\right]}_{0}}{{\left[S\right]}_{0}+K_{M}}$$

where *υ*_0_ is the initial reaction rate, [*S*]_0_ is the initial substrate concentration, *V*_max_ is the maximum reaction rate, and *K*_M_ is the Michaelis–Menten constant. *υ*_0_ and [*S*]_0_ are the dependent and independent variables, respectively, and *K*_m_ and *V*_max_ are the fitted parameters. The lower limit for *K*_M_ determination was set to 10 μM, below which the activity was considered as not determined. The *V*_max_ */K*_M_ parameters were calculated and averaged; the values can be found in Table S2. The ratio of signals at 222 nm derived from CD spectra was used for normalization of activities relative to wild type.

### 4.8 NMR spectroscopy experiments

For the production of isotope‐labeled proteins for NMR experiments, the M9 medium was used with ^15^N ammonium chloride as the sole nitrogen source. Cultures (100 mL) were spun down and resuspended in B‐PER and after 30 min incubation at room temperature, samples were centrifuged, and TROSY‐HSQC spectra of soluble fraction were recorded on a AVIII HD 850 MHz spectrometer (Bruker, Leiderdorp, Netherlands) at 25°C with 6% D_2_O. Data were processed in Topspin 4.1 (Bruker, Leiderdorp, Netherlands). Spectra were analyzed with CCPNmr Analysis V3^62^ software. Peaks of the mutant spectra were assigned where possible by comparison to those in the wild‐type BlaC spectrum and average chemical shift perturbations (CSP), *Δδ*, of the ^1^H (*Δω*_1_) and ^15^N (*Δω*_2_) resonances of backbone amides were calculated using Equation 2.

(2) $$∆\delta =\sqrt{\frac{1}{2}\left({∆\omega }_{1}^{2}+{\left(\frac{∆\omega_{2}}{5}\right)}^{2}\right)}$$

## AUTHOR CONTRIBUTIONS

**Aleksandra Chikunova:** Conceptualization (supporting); investigation (lead); methodology (lead); validation (lead); visualization (equal); writing – original draft (equal). **Marcellus Ubbink:** Conceptualization (lead); funding acquisition (lead); methodology (supporting); project administration (lead); supervision (lead); validation (supporting); visualization (equal); writing – original draft (equal).

## CONFLICT OF INTEREST

The authors declare that they have no conflicts of interest with the contents of this article.

## Supporting information


**Data S1:** Chikunova_et_al_supplementary data.pdf:
**Supplementary Tables**: BlaC sequence with ConSurf grades; Data from in‐cell and *in vitro* experiments
**Supplementary Figures**: Gels displaying soluble fractions of cell cultures expressing wild‐type and mutant BlaC; Example of CD spectra; Nitrocefin kinetic curves; Thermal shift assay data; Examples of NMR spectra of cell lysates; Localization of BlaC produced by constructs used in this work.
