# Supplementary material for: The roles of highly conserved, non‐catalytic residues in class A β‐lactamases
Source: Protein Sci. 2022 May 17;31(6):e4328. doi: 10.1002/pro.4328 (PMC9112487; doi:10.1002/pro.4328)
Supplement: Supplementary file 1 — Data S1: Chikunova_et_al_supplementary data.pdf: Supplementary Tables: BlaC sequence with ConSurf grades; Data from in‐cell and in vitro experiments Supplementary Figures: Gels displaying soluble fractions of cell cultures expressing wild‐type and mutant BlaC; Example of CD spectra; Nitrocefin kinetic curves; Thermal shift assay data; Examples of NMR spectra of cell lysates; Localization of BlaC produced by constructs used in this work. [file PRO-31-e4328-s001.pdf]

Supplementary materials to

The roles of highly conserved, non-catalytic residues in class A β-lactamases.

A. Chikunova, M. Ubbink

Leiden University, Leiden, The Netherlands

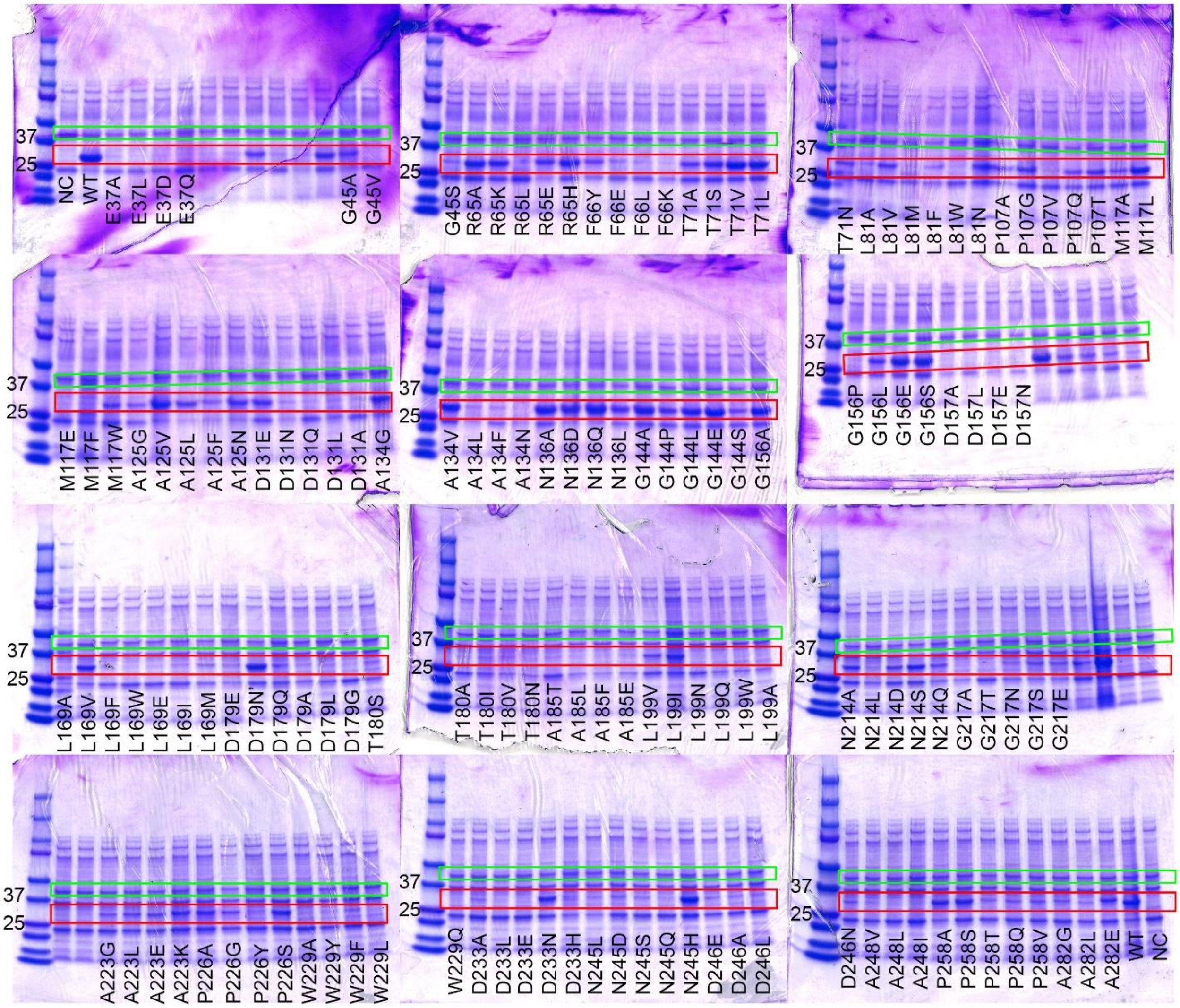

Supplementary Figure 1. Gels displaying soluble fractions of cell cultures expressing WT and mutant BlaC. NC – negative control, cells containing empty vector. Red frame shows the position of BlaC at 31 kDa, green frame shows the position of *E. coli* protein that was used for signal scaling. Non-labeled lanes contain mutants of residues with lower percent conservation, thus not discussed within this study.

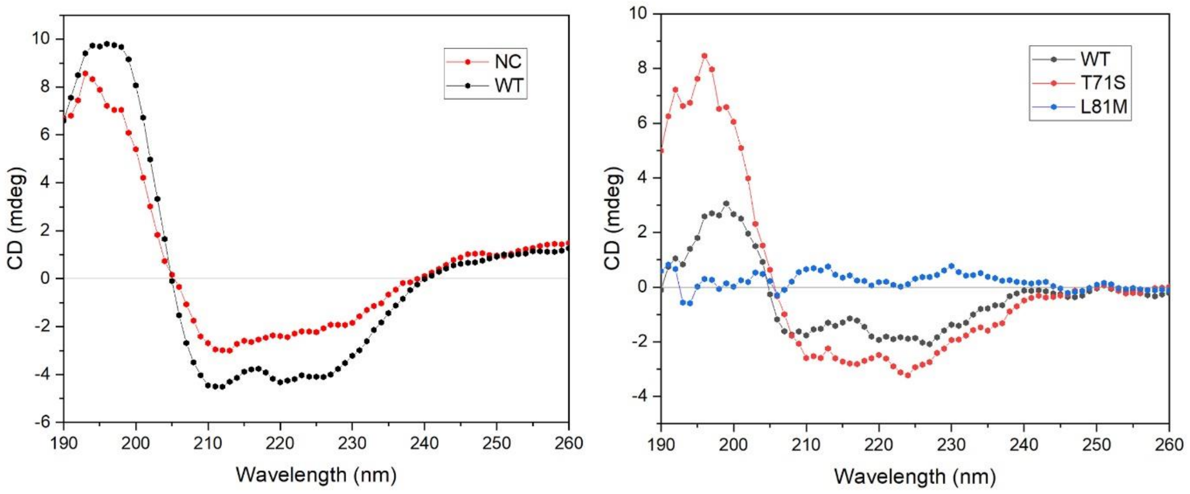

Supplementary Figure 2. Example of CD spectra acquired for negative control (NC) and wild type (WT) (*left panel*); spectra of wild type, a poorly produced mutant (L81M) and a folded mutant (T71S) after background correction (*right panel*).

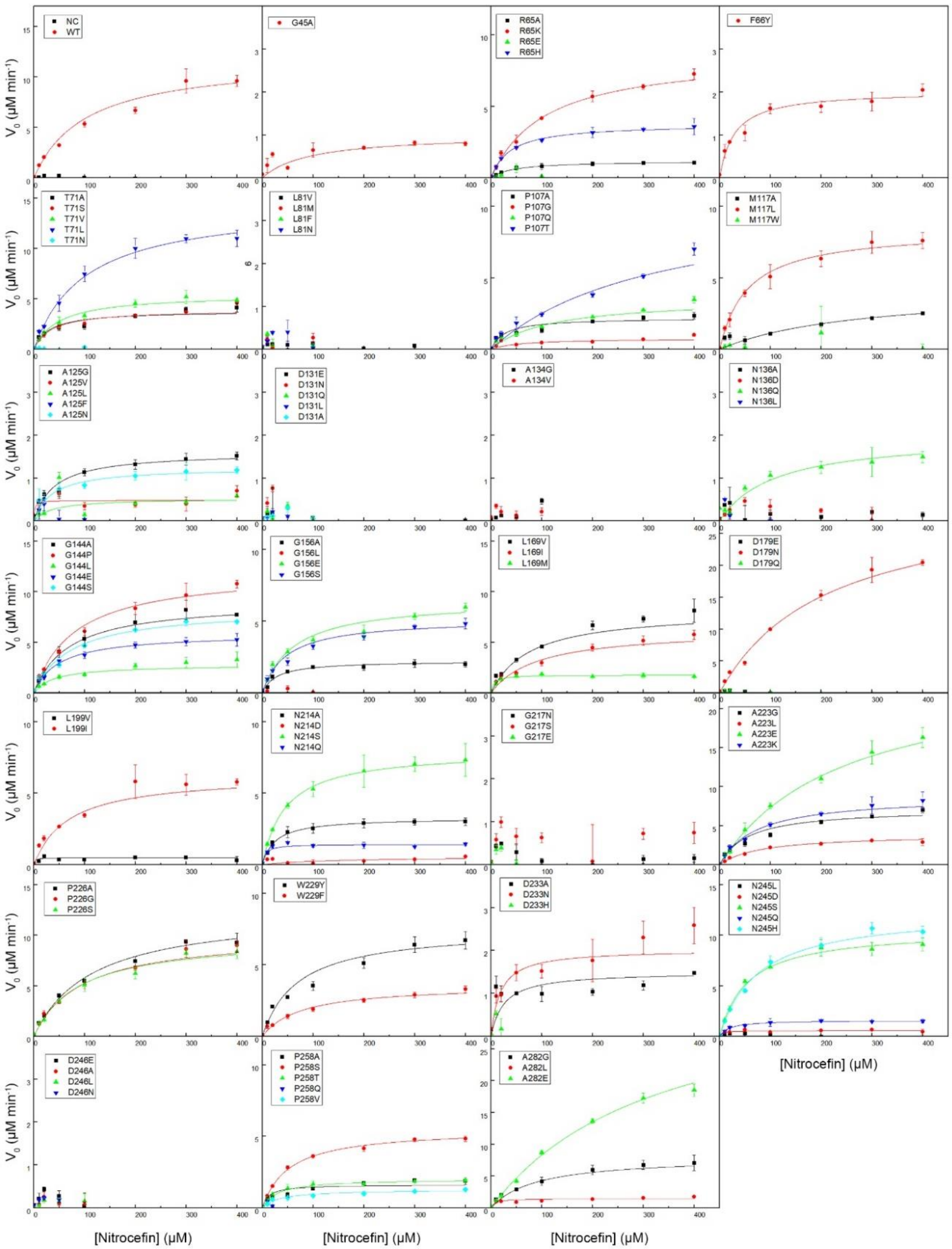

Supplementary Figure 3. Nitrocefin kinetic curves. Error bars represent the standard deviation of a triplicate measurement. Lines represent the Michaelis-Menten fit.

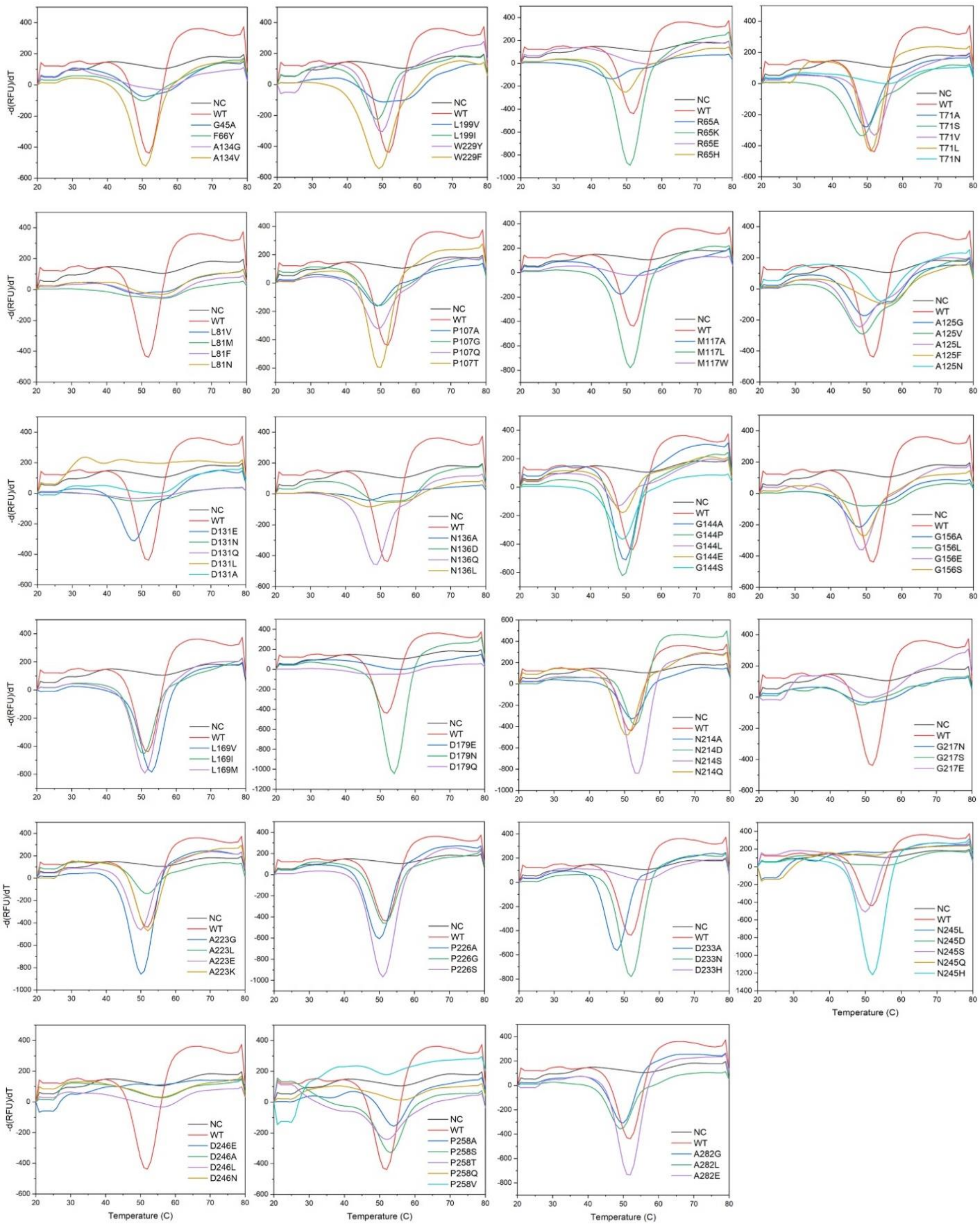

Supplementary Figure 4. Negative derivative of the RFU signal from thermal shift assay for WT and mutant BlaC.

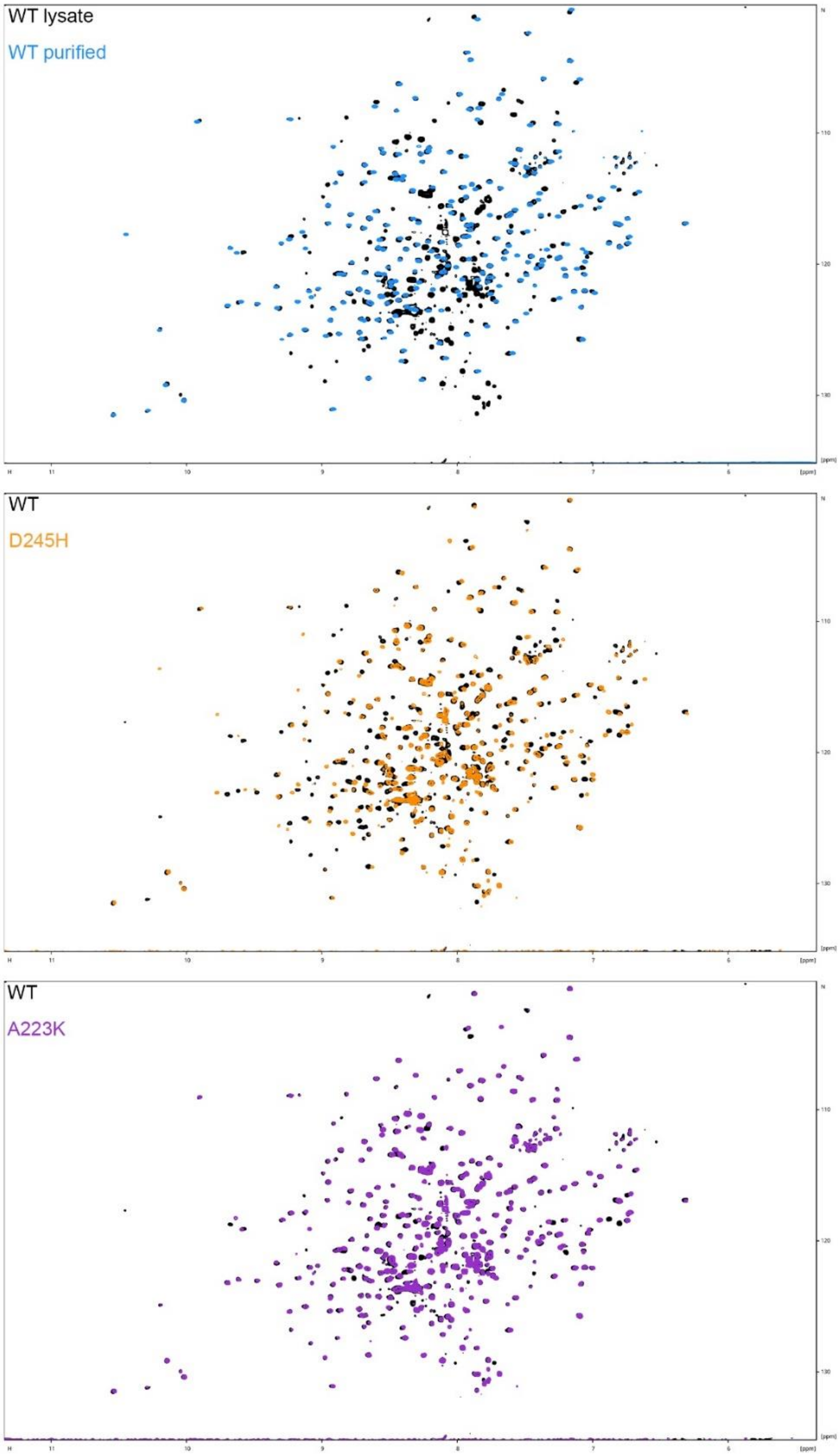

Supplementary Figure 5. Examples of NMR spectra of cell lysates. The upper panel displays overlay of purified wild type in phosphate buffer pH 6.4 (in blue) and the soluble fraction of cell lysate of a culture expressing the wild type *blaC* gene (in black). The middle and bottom panels display the overlays of the cell lysate spectra of the second-shell mutant D245H (in orange) with wild type BlaC (in black) and of the third-shell mutant A223K (in purple) with wild type BlaC (in black).

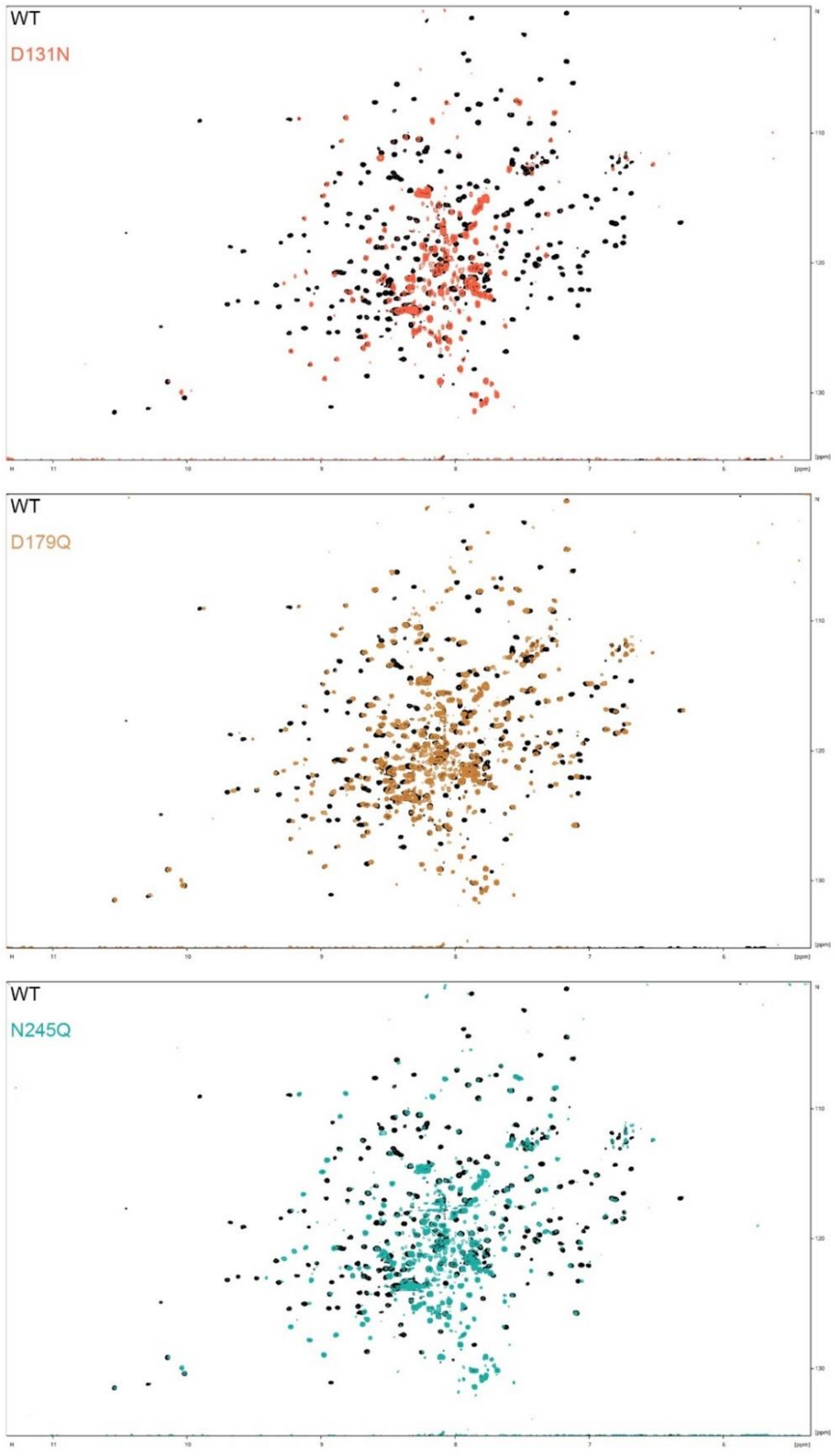

Supplementary Figure 6. Examples of NMR spectra of cell lysates with unfolded protein. The panels display the overlays of spectra of the soluble fraction of cell lysates of mutants D131N (in pink, top), D179Q (in brown, middle) and N245Q (in turquoise, bottom) with that of wild type BlaC (in black).

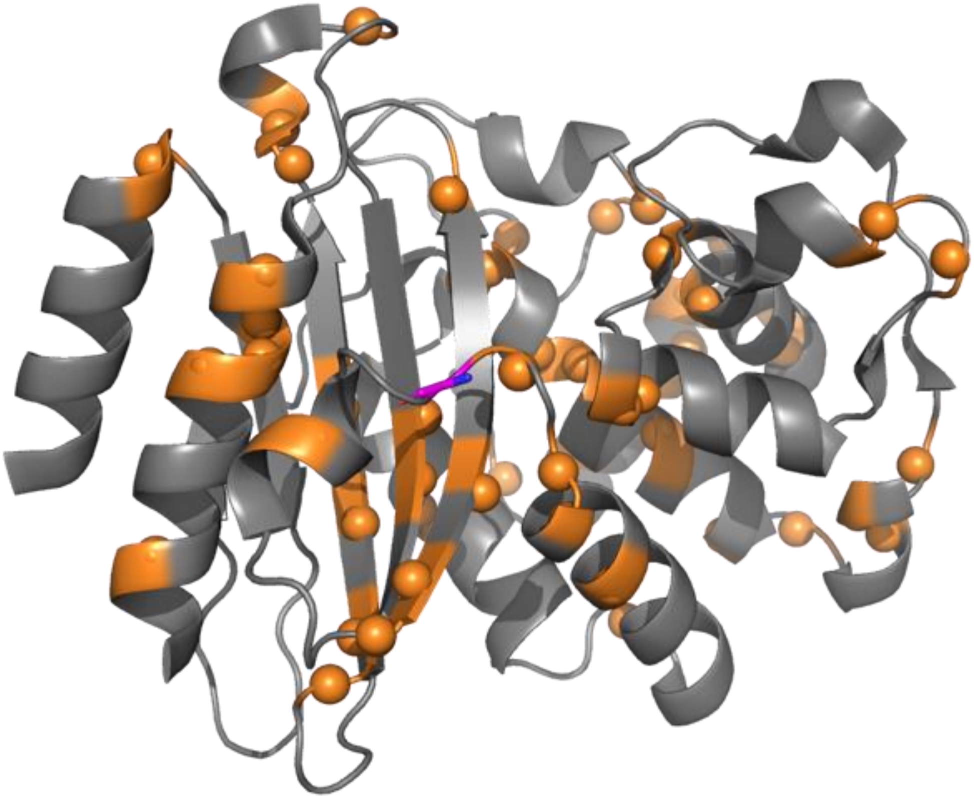

Supplementary Figure 7. Example of the location of nuclei with significant CSP in a second-shell mutant (G217S) located on the protein surface. The mutated residue is shown in magenta sticks.

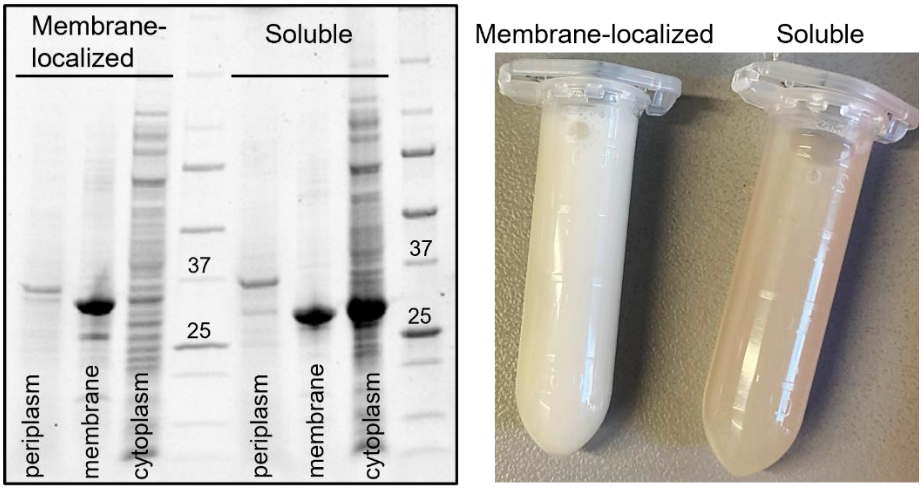

Supplementary Figure 8. Detection of BlaC location after overexpression with and without a signal peptide for membrane localization. Most protein is found in membrane in construct with the signal peptide, which is shown on gel (left) and by the lighter color of the cells (right). Most protein is found in cytoplasm after expression in construct without the signal peptide with some protein detected in the membrane fraction, which can be possibly attributed to inclusion bodies formation due to overexpression (membrane fraction and inclusion bodies were not separated).

Supplementary Table 1. BlaC sequence with ConSurf grades and percentages of the most occurring residue per position.

Position (Ambler)	ConSurf Grade	Residue in BlaC	The most occurring residue (%)
28	1	Asp	D (29.48)
29	1	Leu	L (40.417)
30	1	Ala	DE (16.242)
31	1	Asp	R (17.812)
32	1	Arg	E (21.495)
33	5	Phe	F (50.947)
34	1	Ala	A (44.05)
35	1	Glu	E (24.33)
36	8	Leu	L (88.525)
37	9	Glu	E (99.187)
38	1	Arg	R (29.268)
39	1	Arg	K (23.984)
40	2	Tyr	F (32.657)
41	2	Asp	G (43.205)
42	8	Ala	A (55.263)
43	8	Arg	R (89.879)
44	8	Leu	L (92.308)
45	9	Gly	G (100)
46	8	Val	V (87.247)
47	5	Tyr	Y (48.583)
48	7	Val	A (69.636)
49	2	Pro	L (38.945)
50	7	Ala	D (76.316)
51	7	Thr	T (85.366)
52	1	Gly	G (54.268)
53	5	Thr	T (57.317)
54	6	Thr	G (66.667)
55	2	Ala	R (39.676)
56	3	Ala	T (37.652)
57	3	Ile	V (46.356)
59	1	Glu	A (32.186)
60	4	Tyr	Y (56.68)
61	8	Arg	R (85.02)
62	3	Ala	A (45.344)
63	4	Asp	D (59.312)
64	8	Glu	E (89.069)

Position (Ambler)	ConSurf Grade	Residue in BlaC	The most occurring residue (%)
65	9	Arg	R (96.154)
66	9	Phe	F (99.798)
67	7	Ala	A (57.895)
68	6	Phe	Y (29.96)
69	6	Cys	C (54.453)
70	9	Ser	S (100)
71	9	Thr	T (98.178)
72	7	Phe	F (71.862)
73	9	Lys	K (100)
74	4	Ala	A (51.215)
75	5	Pro	L (65.182)
76	7	Leu	A (68.016)
77	6	Val	A (65.385)
78	6	Ala	A (55.466)
79	6	Ala	A (73.684)
80	5	Val	V (60.324)
81	9	Leu	L (99.19)
82	2	His	Q (21.457)
83	4	Gln	R (37.854)
86	5	Asn	S (21.837)
87	5	Pro	S (25.562)
88	1	Leu	L (20.654)
89	2	Thr	E (33.266)
90	2	His	L (21.053)
91	7	Leu	L (85.83)
92	1	Asp	D (49.798)
93	3	Lys	R (35.223)
94	5	Leu	R (46.154)
95	5	Ile	I (56.275)
96	1	Thr	T (39.676)
97	7	Tyr	Y (82.591)
98	1	Thr	T (33.603)
99	1	Ser	K (30.567)
100	1	Asp	D (32.996)
101	7	Asp	D (83.401)
102	7	Ile	L (81.174)

103	6	Arg	V (69.636)
104	1	Ser	T (22.065)
105	4	Ile	Y (64.372)
106	8	Ser	S (77.53)
107	9	Pro	P (98.583)
108	5	Val	V (45.749)
109	8	Ala	T (88.462)
110	6	Gln	E (71.255)
111	4	Gln	K (59.919)
112	7	His	H (81.579)
113	5	Val	V (69.98)
114	1	Gln	D (35.628)
115	1	Thr	T (50.405)
116	8	Gly	G (94.13)
117	9	Met	M (92.51)
118	6	Thr	T (76.316)
119	6	Ile	L (52.227)
120	3	Gly	A (32.794)
121	5	Gln	E (61.538)
122	7	Leu	L (80.567)
123	5	Cys	C (69.636)
124	6	Asp	D (38.259)
125	9	Ala	A (99.798)
126	7	Ala	A (71.862)
127	4	Ile	I (41.498)
128	8	Arg	R (47.773)
129	3	Tyr	Y (63.968)
130	9	Ser	S (100)
131	9	Asp	D (100)
132	9	Gly	N (92.308)
133	8	Thr	T (81.579)
134	9	Ala	A (99.393)
135	6	Ala	A (49.19)
136	9	Asn	N (100)
137	8	Leu	L (87.045)
138	7	Leu	L (76.721)
139	6	Leu	L (61.741)
140	2	Ala	K (24.899)
141	2	Asp	E (23.887)
142	4	Leu	L (62.753)
143	8	Gly	G (95.344)
144	9	Gly	G (98.988)
145	8	Pro	P (94.737)
145A	1	Gly	A (31.377)
145B	1	Gly	G (53.239)
145C	6*	Gly	G (100)
145D	6*	Thr	T (100)
146	6*	Ala	A (100)
147	6*	Ala	A (100)
148	5	Phe	L (42.915)
149	7	Thr	T (55.061)
150	3	Gly	A (44.332)
151	3	Tyr	F (35.02)
152	6	Leu	L (55.668)
153	7	Arg	R (82.794)
154	2	Ser	S (37.247)
155	4	Leu	I (46.964)
156	9	Gly	G (97.773)
157	9	Asp	D (99.19)
158	1	Thr	D (20.445)
159	1	Val	V (50.405)
160	8	Ser	T (63.968)
161	7	Arg	R (67.004)
162	7	Leu	L (53.846)
163	7	Asp	D (75.101)
164	9	Ala	R (92.713)
165	1	Glu	W (23.887)
166	9	Glu	E (99.595)
167	6	Pro	P (61.134)
168	3	Glu	E (51.417)
169	9	Leu	L (94.939)
170	9	Asn	N (87.449)
171	4	Arg	E (37.045)
172	7	Asp	A (61.943)
173	1	Pro	I (26.978)
174	8	Pro	P (94.523)
175	6	Gly	G (87.652)
176	8	Asp	D (81.579)
177	1	Glu	P (39.474)
178	8	Arg	R (88.057)
179	9	Asp	D (99.798)
180	9	Thr	T (99.595)

181	8	Thr	T (58.3)	222	8	Arg	R (92.713)
182	8	Thr	T (89.271)	223	9	Ala	A (96.964)
183	8	Pro	P (92.308)	224	8	Gly	G (92.51)
184	2	His	R (35.83)	225	6	Phe	V (50.405)
185	9	Ala	A (94.332)	226	9	Pro	P (98.381)
186	7	Ile	M (48.178)	227	1	Ala	A (26.735)
187	6	Ala	A (71.66)	228	1	Asp	G (57.287)
188	1	Leu	A (24.089)	229	9	Trp	W (97.976)
189	5	Val	S (41.296)	230	1	Lys	V (19.028)
190	6	Leu	L (74.291)	231	8	Val	V (88.664)
191	4	Gln	R (38.866)	232	6	Ile	G (66.802)
192	3	Gln	A (39.271)	233	9	Asp	D (96.761)
193	4	Leu	L (47.976)	234	9	Lys	K (98.178)
194	3	Val	V (35.425)	235	9	Thr	T (85.223)
195	6	Leu	L (75.709)	236	9	Gly	G (100)
196	6	Gly	G (87.247)	237	5	Thr	T (35.425)
197	3	Asn	D (62.955)	238	7	Gly	G (87.652)
198	3	Ala	A (57.49)	240	2	Asp	D (31.984)
199	9	Leu	L (100)	241	6	Tyr	Y (77.393)
200	1	Pro	P (34.615)	242	8	Gly	G (95.142)
201	1	Pro	A (24.494)	243	7	Arg	T (67.206)
202	1	Asp	A (29.352)	244	6	Ala	R (41.903)
203	7	Lys	K (31.377)	245	9	Asn	N (92.51)
204	8	Arg	R (90.283)	246	9	Asp	D (96.356)
205	1	Ala	A (30.972)	247	7	Ile	I (68.826)
206	4	Leu	Q (39.676)	248	8	Ala	A (74.494)
207	8	Leu	L (91.093)	249	6	Val	V (67.004)
208	1	Thr	T (35.425)	250	3	Val	V (37.652)
209	1	Asp	D (41.7)	251	7	Trp	W (93.725)
210	8	Trp	W (91.903)	252	8	Ser	P (85.425)
211	6	Met	L (62.955)	254	4	Pro	P (74.696)
212	2	Ala	K (29.96)	255	1	Thr	D (22.267)
213	5	Arg	G (42.105)	256	2	Gly	R (42.105)
214	9	Asn	N (92.915)	257	4	Val	A (49.393)
215	6	Thr	T (63.968)	258	9	Pro	P (97.976)
216	9	Thr	T (94.737)	259	4	Tyr	I (56.68)
217	9	Gly	G (94.332)	260	6	Val	V (65.385)
218	2	Ala	D (57.085)	261	4	Val	L (40.688)
219	1	Lys	A (23.732)	262	6	Ala	A (71.197)
220	8	Arg	R (45.842)	263	5	Val	V (51.619)
221	8	Ile	I (72.672)	264	7	Met	Y (42.51)

265	7	Ser	S (48.785)
266	6	Asp	T (44.332)
267	6	Arg	R (40.486)
268	1	Ala	D (23.887)
269	1	Gly	G (50)
270	4*	Gly	G (83.333)
271	1	Gly	T (20.445)
272	2	Tyr	K (33.266)
273	3	Asp	D (60.041)
274	6	Ala	A (80.652)
275	1	Glu	E (24.224)
276	1	Pro	Y (30.608)
277	4	Arg	D (40.592)
278	5	Glu	D (40.764)
279	4	Ala	A (41.957)
280	6	Leu	L (55.58)
281	6	Leu	I (60.659)
282	9	Ala	A (94.222)
283	2	Glu	E (41.111)
284	7	Ala	A (69.265)
285	6	Ala	A (53.452)
286	4	Thr	R (36.748)
287	2	Cys	IV (28.285)
288	4	Val	V (59.821)
289	3	Ala	A (32.265)
290	1	Gly	D (16.898)
291	1	Val	A (43.72)
292	4	Leu	L (70.812)
293	2	Ala	G (32.87)

Supplementary Table 2. Data from *in vivo* and *in vitro* experiments. The orange and purple color represent the second and the third shell respectively. Color coding for the experimental data is as follows: green – (almost) as good as wild type, yellow – somewhat worse than wild type, red – considerably worse than wild type. ND – not detected.

Conserved residue	Mutations	MIC (μg mL^{-1})		Protein on gel		Nitrocefin reaction in cells		T_m	NMR	V_{max}/K_M in cell lysate (10^{-2} min^{-1})	CD signal at 222 nm relative to WT	Relative activity
		Ampicillin	Carbenicillin	Lysate	Supernatant (Relative to WT)	Whole cell (within 10 min)	Supernatant (first minutes)					
	WT	100	1000		1			52	Folded protein	8.9 ± 0.3	1	1
	NC	3	20		ND			ND	Only *E. coli* protein peaks	ND	Used as background	ND
Glu37	E37A	3	20		0.01							
	E37L	3	20		ND							
	E37D	3	20		ND							
	E37Q	3	20		ND							
Gly45	G45A	3	20		0.18			51	Low signal	3.5 ± 1.8	1.0	0.4
	G45V	3	20		ND							
	G45S	3	20		0.12							
Arg65	R65A	3	20		0.67			46	Folded protein in low quantity	2.9 ± 0.9	0.4	0.7
	R65K	40	200		0.90			51	Folded protein	7.9 ± 0.7	2.0	0.4
	R65L	3	20		ND							
	R65E	3	20		1.04			ND	Low signal	ND	1.1	ND
	R65H	5	50		1.76			50	Folded protein	9.3 ± 0.9	1.0	1.1
Phe66	F66Y	3	20		0.99			50	Low signal	6.5 ± 1.2	0.9	0.8
	F66E	3	20		0.17							
	F66L	3	20		0.07							
	F66K	3	20		0.05							
Thr71	T71A	30	200		0.42			50	Folded protein	9.0 ± 0.2	0.9	1.1

	T71S	3	20		0.51			49	Folded protein in low quantity	8.2 ± 0.7	1.5	0.6
	T71V	80	1000		1.06			52	Folded protein	10.0 ± 0.2	1.0	1.1
	T71L	80	1000		0.92			51	Folded protein	15.6 ± 0.3	3.1	0.6
	T71N	3	20		0.43			ND	Folded and unfolded protein	ND	0.5	ND
	L81A	3	20		0.09							
	L81V	3	20		0.37			49	Folded and unfolded protein	ND	0.4	ND
Leu81	L81M	30	200		0.05			ND	Unfolded protein	ND	ND	ND
	L81F	3	20		0.28			ND	Low signal	ND	ND	ND
	L81W	3	20		0.07							
	L81N	3	20		0.50			ND	Low signal	ND	ND	ND
	P107A	10	200		0.71			49	Folded protein	5.8 ± 0.2	0.8	0.8
	P107G	10	200		0.32			50	Low signal	3.4 ± 0.1	0.9	0.4
Pro107	P107V	10	200		0.03							
	P107Q	10	200		0.45			49	Folded protein	2.7 ± 0.05	1.4	0.2
	P107T	10	200		0.35			50	Folded protein	2.9 ± 0.2	1.0	0.3
	M117A	3	20		0.49			48	Folded protein in low quantity	2.4 ± 0.3	1.7	0.2
	M117L	80	1000		1.18			51	Folded protein	14.0 ± 1.9	2.1	0.7
Met117	M117E	3	20		ND							
	M117F	3	20		ND							
	M117W	3	20		0.38			ND	Low signal	ND	0.7	ND
	A125G	3	20		0.40			49	Low signal	3.7 ± 0.6	0.9	0.4
	A125V	3	20		1.33			49	Folded protein	ND	1.3	ND
Ala125	A125L	3	20		1.17			48	Folded protein	4.7 ± 1.2	1.1	0.5
	A125F	3	20		0.41			ND	Low signal	ND	0.2	ND
	A125N	3	20		0.73			ND	Folded and unfolded protein	3.9 ± 0.8	0.6	0.7
	D131E	3	20		0.56			48	Folded and unfolded protein	ND	1.0	ND
Asp131	D131N	3	20		0.10			49	Folded and unfolded protein	ND	ND	ND
	D131Q	3	20		0.04			48	Low signal	ND	ND	ND

	D131L	3	20		0.05			ND	Low signal	ND	ND	ND
	D131A	3	20		ND			ND	Low signal	ND	ND	ND
Ala134	A134G	3	20		0.62			ND	Folded protein in low quantity	ND	0.7	ND
	A134V	3	20		1.15			51	Folded protein	ND	2.0	ND
	A134L	3	20		0.12							
	A134F	3	20		0.12							
	A134N	3	20		ND							
Asn136	N136A	5	200		1.44			47	Folded protein	ND	ND	ND
	N136D	3	20		1.92			50	Folded and unfolded protein	ND	0.9	ND
	N136Q	3	100		2.10			49	Folded protein	2.3 ± 0.3	0.7	0.4
	N136L	3	20		1.48			47	Low signal	ND	ND	ND
Gly144	G144A	5	200		1.35			50	Folded protein	13.0 ± 2.0	2.2	0.7
	G144P	5	100		0.98			49	Folded protein	12.0 ± 0.7	2.1	0.6
	G144L	3	20		1.24			48	Folded protein	5.0 ± 1.4	1.5	0.4
	G144E	15	200		1.90			49	Folded protein	12.0 ± 3.0	1.8	0.7
	G144S	15	200		0.73			49	Folded protein	10.0 ± 1.3	1.4	0.8
Gly156	G156A	3	20		1.26			48	Folded protein	6.7 ± 1.1	1.0	0.7
	G156P	3	20		ND							
	G156L	3	20		0.67			49	Low signal	ND	0.3	ND
	G156E	3	20		1.50			49	Folded protein	10.0 ± 2.4	1.7	0.7
	G156S	3	20		1.25			49	Folded protein	8.4 ± 1.0	1.1	0.9
Asp157	D157A	3	20		ND							
	D157L	3	20		ND							
	D157E	3	20		ND							
	D157N	3	20		ND							
Leu169	L169A	5	20		ND							
	L169V	40	200		0.68			53	Folded and unfolded protein	10.0 ± 2.0	1.9	0.6
	L169F	3	20		0.14							

	L169W	3	20		ND							
	L169E	3	20		ND							
	L169I	40	200		0.16			51	Folded protein	6.0 ± 2.0	1.5	0.4
	L169M	30	200		0.47			51	Folded protein	ND	2.2	ND
Asp179	D179E	5	20		0.14			ND	Folded and unfolded protein	ND	0.5	ND
	D179N	100	2000		1.41			54	Folded protein	14.0 ± 1.1	3.5	0.4
	D179Q	5	20		0.36			48	Folded and unfolded protein	ND	ND	ND
	D179A	10	200		0.16							
	D179L	5	20		0.12							
	D179G	5	100		ND							
Thr180	T180S	40	500		0.17							
	T180A	3	20		ND							
	T180I	3	20		0.13							
	T180V	3	20		0.16							
	T180N	3	20		ND							
Ala185	A185T	3	20		0.10							
	A185L	3	20		ND							
	A185F	3	20		ND							
	A185E	5	100		0.12							
Leu199	L199V	3	20		0.61			50	Folded and unfolded protein	ND	ND	ND
	L199I	3	50		0.43			48	Folded protein	10.0 ± 0.6	2.1	0.5
	L199N	3	20		ND							
	L199Q	3	20		ND							
	L199W	3	20		ND							
	L199A	3	20		ND							
Asn214	N214A	100	1000		0.63			52	Folded protein	14.0 ± 1.5	0.8	2.0
	N214L	3	20		ND							
	N214D	3	20		0.55			53	Folded protein	ND	2.3	ND

	N214S	100	1000		0.93			54	Folded protein	17.0 ± 2.9	1.9	1.0
	N214Q	30	200		0.59			51	Folded protein	ND	2.0	ND
Gly217	G217A	3	20		ND							
	G217T	3	20		ND							
	G217N	3	20		0.23			50	Folded protein	ND	0.8	ND
	G217S	3	20		0.39			49	Folded protein	ND	1.0	ND
	G217E	3	20		0.42			51	Folded protein	ND	0.6	ND
Ala223	A223G	30	500		0.29			50	Folded protein	8.5 ± 1.1	2.0	0.5
	A223L	30	500		0.41			52	Folded protein	5.1 ± 0.5	0.3	1.7
	A223E	30	500		0.45			50	Folded protein	10.0 ± 1.4	2.9	0.4
	A223K	60	1000		0.68			52	Folded protein	11.0 ± 1.4	1.3	0.9
Pro226	P226A	10	200		0.85			50	Folded protein	11.0 ± 0.8	2.5	0.5
	P226G	30	500		0.57			52	Folded protein	9.8 ± 0.5	1.3	0.8
	P226Y	3	20		ND							
	P226S	10	200		1.01			51	Folded protein	9.7 ± 0.6	2.2	0.5
Trp229	W229A	3	20		ND							
	W229Y	3	50		0.13			50	Folded protein in low quantity	8.0 ± 0.3	0.9	1
	W229F	3	50		0.12			49	Folded protein in low quantity	4.3 ± 0.5	1.5	0.3
	W229L	3	20		ND							
	W229Q	3	20		ND							
Asp233	D233A	10	200		0.16			48	Folded protein	ND	2.2	ND
	D233L	3	20		ND							
	D233E	3	20		ND							
	D233N	100	500		0.71			52	Folded protein	9.3 ± 2.4	2.4	0.4
	D233H	10	200		ND			ND	Folded protein in low quantity	ND	0.7	ND
Asn245	N245L	3	20		ND			ND	Unfolded protein	ND	ND	ND
	N245D	3	20		0.16			ND	Folded protein	ND	0.8	ND
	N245S	15	200		0.16			50	Folded protein	21.0 ± 1.0	2.4	1.0

	N245Q	3	20		0.10			45	Folded and unfolded protein	7.8 ± 0.6	0.7	1.3
	N245H	100	2000		0.93			52	Folded protein	16.0 ± 0.5	3.6	0.5
Asp246	D246E	3	20		0.18			ND	Folded protein in low quantity	ND	0.2	ND
	D246A	3	20		0.16			ND	Folded protein in low quantity	ND	0.6	ND
	D246L	3	20		0.11			ND	Low signal	ND	0.5	ND
	D246N	3	20		0.20			ND	Folded protein in low quantity	ND	0.9	ND
Ala248	A248V	3	20		ND							
	A248L	5	50		ND							
	A248I	3	20		ND							
Pro258	P258A	80	1000		0.55			54	Folded protein in low quantity	5.5 ± 1.3	0.4	1.7
	P258S	80	1000		0.70			53	Folded protein	11.0 ± 0.1	1.0	1.3
	P258T	10	200		0.28			52	Low signal	8.7 ± 2.0	0.7	1.5
	P258Q	15	200		0.22			ND	Low signal	ND	0.3	ND
	P258V	5	50		0.27			ND	Low signal	3.4 ± 1.1	0.7	0.6
Ala282	A282G	30	500		0.23			50	Folded protein in low quantity	9.7 ± 0.6	1.5	0.7
	A282L	5	50		0.17			49	Folded protein in low quantity	ND	0.9	ND
	A282E	80	1000		0.72			52	Folded protein	11.0 ± 0.5	3.2	0.4
